# Sequence-Anchored Shared Tumor-Specific Epitopes for Pre-Manufactured HLA-Matched mRNA Cancer Vaccine Libraries: A Pan-Cancer Framework

**DOI:** 10.3390/biom16071015

**Published:** 2026-07-11

**Authors:** Sarfaraz K. Niazi

**Affiliations:** College of Pharmacy and Pharmaceutical Sciences, Washington State University, Spokane, WA 99202, USA; s.niazi@wsu.edu

**Keywords:** shared tumor-specific epitope, pre-manufactured mRNA cancer vaccine, HLA-matched vaccine library, viral oncoprotein, mutant KRAS, minimal residual disease

## Abstract

A single vaccine cannot prevent or treat all cancers; however, recurrent tumor-specific epitopes may facilitate the development of pre-manufactured, HLA-matched mRNA vaccines tailored for specific molecular subgroups. We define the shared tumor-specific epitope as a recurring peptide derived from a viral oncoprotein, a driver mutation, a frameshift, an altered protein C-terminus, or a fusion junction, and we employ a rigorous cancer-cell-only criterion: a target must be recurrent within a defined subgroup, absent from essential normal tissues at the peptide–HLA level, naturally presented on tumor cells, and sufficiently clonal to minimize immune escape. Under this criterion, we present fifteen sequence-anchored reference designs alongside one conceptual placeholder across thirteen candidates divided into four superclasses: viral oncoproteins (such as HPV16/18 E6 and E7 as attenuated antigenic reference designs; Merkel cell polyomavirus serving as a design-specific placeholder), recurrent driver neoepitopes (including KRAS G12/G13, IDH1 R132H, and H3 K27M), hematologic neoantigens (such as NPM1 Type A C-terminus; and a single CALR exon 9 construct encoding the shared novel C-terminus of types 1 and 2 mutations), and fusion junctions (notably EWS-FLI1 and BCR-ABL). Each open reading frame is anchored to a canonical accession with its documented event; representative ORFs are provided as reference designs, with the intended residue-level verification records. These sequence designs are intended as reference constructs and are not suitable as clinical-grade or manufacturing-ready products; they require independent residue-level validation and comprehensive safety assessments prior to laboratory or clinical application. The historical record of non-personalized vaccination—including HPV and hepatitis B prophylaxis, intravesical BCG, and unsuccessful tumor-associated antigen trials—frames both the potential and limitations of such approaches. The practical product is not a universal vaccine but rather a governed library aligned with specific genotype, viral etiology, HLA context, and clinical setting. Currently, none of these designs have established proof-of-benefit-tier evidence.

## 1. Introduction and Conceptual Framing

The proposition that a single vaccine could prevent or treat all cancers is not supported by the biology of malignant transformation. Most cancers arise through combinations of somatic mutations, copy-number changes, epigenetic states, and immune-evasion strategies, and the antigens presented to the immune system reflect that diversity. A defensible question is whether recurrent tumor-specific epitopes exist that can support pre-manufactured, HLA-matched mRNA cancer vaccines for defined molecular subgroups, while reserving personalized neoantigen vaccines for settings in which shared targets are insufficient.

This manuscript adopts the phrase “shared tumor-specific vaccination” rather than “generic cancer vaccination” and uses “pre-manufactured” in place of “off-the-shelf” wherever the precision matters, since the products described are held in inventory but are selected per patient by tumor genotype and HLA type. In infectious disease vaccinology, conservation refers to the stability of sequences across pathogen strains. In cancer, the relevant properties are recurrence across patients, clonality within a tumor, and absence from essential normal tissue. The standard adopted herein is more conservative than the National Cancer Institute antigen-prioritization framework [1], which included several tumor-associated antigens also present in normal tissues. That framework remains a useful historical reference but should not be read as endorsing a strict tumor-only criterion.

A non-personalized cancer vaccine is credible only when the target population is explicitly defined. Practical examples include HPV16-positive head and neck squamous cell carcinoma; KRAS G12D-mutant pancreatic ductal adenocarcinoma in a patient whose HLA genotype is predicted to present the relevant mutant peptide; mismatch-repair-deficient Lynch-associated neoplasia; IDH1 R132H glioma; and NPM1-mutated acute myeloid leukemia in molecular remission. Inherited risk syndromes require particular attention, because a risk allele may be present in normal tissues long before any malignant cells emerge. A risk gene identifies individuals to monitor; a tumor-specific epitope defines which immune response can be safely mobilized. Because the target classes are defined by molecular events rather than by tissue of origin, individual constructs are inherently cross-cancer: a given KRAS hotspot, HPV E6/E7, or EBV latent-antigen module is applicable to every histology that recurrently carries that event. The library is therefore organized by shared target rather than by single cancer type, and the term cancer vaccine library is used here in this broad, event-defined sense rather than implying a vaccine restricted to one tumor site.

**Figure 1:**
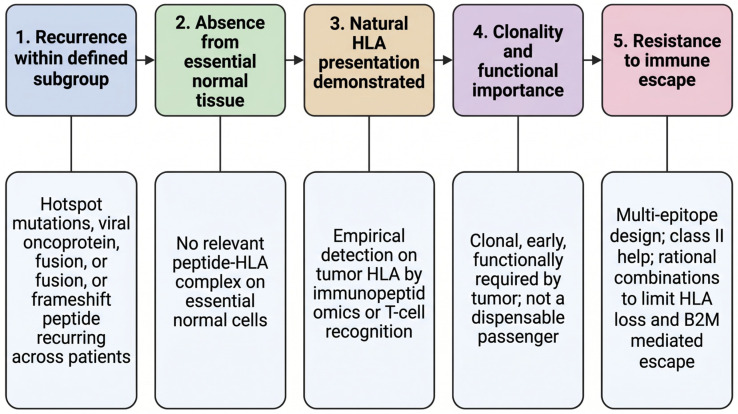
Strict Target Filter. Sequential filtering of candidate antigens by recurrence within a defined subgroup; absence from essential normal tissues at the peptide–HLA level; verified natural presentation on tumor HLA molecules; clonality and functional relevance within the tumor; and resistance to immune escape. Recurrence alone is insufficient; exclusion from normal tissues and confirmed presentation are required criteria. Created in BioRender. Niazi, S. (2026) https://BioRender.com/u6t8q34.

Two shortcuts are dismissed throughout. First, not every recurrent mutation produces a usable epitope; many mutations are neither processed, presented, nor recognized. Second, several familiar cancer antigens, including HER2, MUC1, hTERT, WT1, survivin, prostate-specific membrane antigen (PSMA), mammaglobin, and alpha-lactalbumin, are tumor-associated, tissue-restricted, lineage-specific, developmental, or context-dependent rather than strictly cancer-exclusive. They may have value in specific therapeutic or research settings, but they do not, in themselves, substantiate the shared tumor-specific vaccine hypothesis (Figure 1).

## 2. Historical Context: What Non-Personalized Vaccination Has Shown

Prior to expanding the shared tumor-specific framework to mRNA technology, historical data indicate which targets and disease contexts have demonstrated responsiveness to immunization, and which have not. Furthermore, it demonstrates that platform selection cannot mitigate the disadvantages of biologically unsuitable targets.

### 2.1. Preventive Vaccination Against Oncogenic Infection

The most reproducible successes of cancer vaccines have been against the infections that cause cancer. Cancers attributable to infection account for a substantial fraction of global incidence, with human papillomavirus (HPV), *Helicobacter pylori*, hepatitis B, and hepatitis C as the principal causative agents [2]. Universal infant hepatitis B vaccination in Taiwan was followed by a decline in childhood hepatocellular carcinoma [3], and a twenty-year follow-up confirmed a sustained reduction in liver cancer incidence within vaccinated cohorts [4]. The general principle is that disrupting an upstream cause of cancer prevents cancer at the population level.

HPV vaccination has reinforced this conclusion. A register-based study in England documented substantial reductions in cervical cancer and CIN3 incidence following the implementation of the national HPV vaccination program, with the most significant declines observed in women vaccinated at ages 12 and 13 (cervical cancer reduced by 87%, 95% CI 72–94%; CIN3 approximately 97% reduction). A nationwide Swedish investigation reported an adjusted incidence rate ratio of 0.37 for invasive cervical cancer among women vaccinated with the quadrivalent HPV vaccine, corresponding to an overall reduction of approximately 63%, and approximately 88% among those vaccinated prior to age 17. Public Health Scotland announced in an agency news release that no cases of cervical cancer had been identified among women who were fully vaccinated at ages 12 to 13 during the period under review. It is important to note that this source is an agency communication and not a peer-reviewed publication; it is cited here solely for completeness. These findings underscore the effectiveness of vaccination prior to viral oncogenesis; however, they do not directly translate into therapeutic cancer vaccination, as the same viral antigens remain present in established invasive tumors, making immune response elicitation more challenging.

### 2.2. Local Immune Therapy: Intravesical BCG in Bladder Cancer

Intravesical Bacillus Calmette–Guérin (BCG) is not an antigen-specific cancer vaccine but is among the most clinically successful vaccine-like immunotherapies in oncology. The European Association of Urology guidance recognizes BCG with maintenance as an effective treatment for reducing recurrence and progression in intermediate- and high-risk non-muscle-invasive bladder cancer [5]. The bladder is anatomically suited to this approach: the mucosa is accessible via the urinary tract, immune stimulation can be administered repeatedly, and endpoints such as recurrence, progression, and cystectomy-free survival are directly observable. In 2024, the U.S. Food and Drug Administration approved nogapendekin alfa inbakicept-pmln (Anktiva), an IL-15 receptor agonist, in combination with BCG for adult patients with BCG-unresponsive carcinoma in situ with or without papillary tumors [6]. The combination strategies outlined here reflect regulatory statuses recorded in the EAU 2026 guidance update [5] and the FDA 2024 Anktiva approval [6], as verified against the cited sources on the access dates listed in the reference list. The relationship between BCG and lung cancer is distinct and far less established. Historical trials and observational analyses explored BCG, given systemically or intrapleurally, as an adjuvant nonspecific immunostimulant in resected lung cancer, but the results were inconsistent and did not establish a reproducible survival benefit, and BCG is not an accepted therapeutic agent for active lung cancer. Some ecological and cohort data have separately suggested a possible association between neonatal BCG vaccination and altered later lung-cancer risk, but these findings are confounded and remain unproven. BCG is therefore included here strictly as a bladder-cancer local-immunotherapy benchmark, not as evidence for a lung-cancer indication. The broader implication is that mature local immune-therapy platforms can be enhanced by incorporating cytokine biology or checkpoint elements rather than being replaced.

### 2.3. Oncolytic-Virus Therapy

Oncolytic viruses function at the intersection of direct tumor destruction and vaccine-like immune priming. They are engineered to selectively target tumor cells, induce cellular lysis, release tumor-associated antigens, provoke local inflammation, and elicit systemic antitumor immune responses. Talimogene laherparepvec (T-VEC, Imlygic) represents the first oncolytic-virus therapy approved by the U.S. Food and Drug Administration (FDA) for the localized treatment of unresectable melanoma lesions in the skin, subcutaneous tissue, and lymph nodes that recur following initial surgical procedures [7]. Its primary application is for injectable lesions, and the existing labeling does not demonstrate a significant improvement in overall survival or an impact on visceral metastases. Historically, oncolytic vaccination has shown efficacy in tumors that are anatomically accessible; however, achieving systemic remission of disseminated disease necessitates more than merely local tumor lysis.

### 2.4. The Boundary Case of Sipuleucel-T

Sipuleucel-T (Provenge) represents the pioneering cellular immunotherapy approved by the FDA, bearing similarities to a therapeutic cancer vaccine. It is indicated for patients with asymptomatic or minimally symptomatic metastatic castration-resistant prostate cancer [8]. This therapy is highlighted due to its influence on shaping expectations for therapeutic vaccination within the field. As an autologous, personalized product derived from the patient’s own immune cells and manufactured specifically for everyone, it falls outside the category of pre-manufactured vaccines discussed in this manuscript. Clinical experience indicates that immune priming against cancer can be both safe and clinically significant, despite limitations such as limited therapeutic efficacy, manufacturing complexity, lack of observable tumor reduction, and competition with other systemic treatments, which have limited its wider adoption. This case also emphasizes the ongoing appeal of prefabricated approaches, offering simpler logistics, quicker availability, and reduced per-patient manufacturing complexity.

### 2.5. Failed Late-Stage Tumor-Associated-Antigen Vaccines

Several late-stage trials of fixed tumor-associated antigen (TAA) vaccines have become cautionary references. The MAGRIT phase 3 trial of the MAGE-A3 cancer immunotherapeutic in resected MAGE-A3-positive non-small-cell lung cancer did not improve disease-free survival versus placebo, and development for that indication was discontinued [9]. Rindopepimut, a fixed peptide vaccine targeting EGFRvIII in glioblastoma, did not improve overall survival in the phase 3 ACT IV trial [10]. PROSTVAC, a viral-vector PSA-targeted vaccine, was safe but did not improve overall survival in metastatic castration-resistant prostate cancer [11]. Tecemotide, a MUC1-targeted vaccine, did not meet its primary overall survival endpoint in patients with unresectable stage III non-small-cell lung cancer in the START trial [12], and updated survival and biomarker analyses did not confirm a benefit [13].

The lesson is not that therapeutic vaccines fail. The reasons for their limited success often include inappropriate target selection and clinical settings: weak self-antigens, single-antigen designs, bulky or poorly selected disease states, inadequate modulation of the immune context, limited biomarker enrichment, and endpoints requiring substantial survival benefits in immunologically resistant diseases. The design principles guiding contemporary programs, such as target foreignness, biomarker selection, multi-antigen configurations, and combinations with checkpoint inhibitors or local immune modulation, are predominantly derived from these inadequacies.

### 2.6. Regional and HLA-Precision Examples

Not all non-personalized therapeutic vaccines have been developed within the regulatory frameworks of the United States or Europe. CIMAvax-EGF, a Cuban vaccine that enhances antibody production against epidermal growth factor, has been developed in Cuba for non-small-cell lung cancer and is available in the United States only through clinical trials conducted at Roswell Park Comprehensive Cancer Center and affiliated sites [14]. The clinical evidence supporting this vaccine is limited and context-specific; CIMAvax has not received approval in the United States, the European Union, or other major regulatory markets, and should not be regarded as broadly validated. OSE2101/Tedopi, a multi-epitope vaccine restricted to HLA-A2-positive patients, exemplifies an HLA-defined precision design. The randomized ATALANTE-1 study compared OSE2101 with chemotherapy in HLA-A2-positive patients with advanced non-small-cell lung cancer who had developed resistance to immunotherapy and reported a survival benefit within a pre-specified subgroup [15]. The results of ATALANTE-1 have not led to global regulatory approval, and OSE2101 should not be considered an established or mature standard comparable to checkpoint inhibitors. This strategy exemplifies a contemporary non-personalized approach: limiting the patient population based on HLA type and treatment context, employing multiple epitopes, and benchmarking against a defined subsequent-line standard. Whether HLA-precision designs will outperform broader approaches remains a hypothesis at this stage, supported by suggestive but inconclusive data rather than confirmed regulatory approval.

## 3. The Cancer-Cell-Only Rule and Target Classes That Survive It

The central rule of this framework is that a proposed target must appear only in malignant or premalignant cells for the intended immune mechanism. For T-cell vaccines, that means not simply low transcript expression in normal tissues but the absence of the relevant peptide–HLA complex on essential normal cells, to the extent that can be demonstrated. This standard is more stringent than the standard often used in therapeutic vaccine discussions and is the appropriate threshold for any claim involving prevention or low-risk contexts, where the tolerance for off-tumor toxicity is much narrower than in metastatic disease (Figure 1).

### 3.1. Viral Oncoproteins

The most prominent target category comprises viral oncoproteins absent from the human genome but expressed because they are necessary for tumor survival. HPV E6 and E7 exemplify this classification; they are not typical human proteins, are recurrently present in HPV-driven malignancies, and facilitate the maintenance of the transformed state. Merkel cell polyomavirus (MCPyV) T antigens belong to the same candidate category, as virus-positive Merkel cell carcinoma exhibits integrated and truncated T antigens that promote tumor growth and are foreign to the host [16]. The development of clinical vaccines targeting MCPyV is less advanced than that for HPV, a disparity that is duly recognized. Vaccines aimed at the MCPyV capsid antigen VP1 are conceptually distinct from T-antigen vaccines, since VP1 does not constitute the primary tumor-maintaining antigen in established Merkel cell carcinoma and should not be regarded as central evidence of shared tumor-specificity. Furthermore, Epstein–Barr virus (EBV) latent antigens, as discussed in Section 9.6, also fall within this category in certain EBV-associated malignancies.

### 3.2. Recurrent Driver Neoepitopes

The second category comprises recurrent driver neoepitopes: mutated residues or mutation-induced peptides absent from the normal proteome. Mutations such as KRAS codons 12 and 13, IDH1 R132H, H3 K27M, and specific fusion junctions satisfy this criterion. Their primary limitation is HLA restriction: a mutated peptide functions as an effective T-cell target only when it is naturally processed and presented by one of the patient’s HLA molecules. The most conclusive evidence that mutant KRAS is immunogenic in vivo derives from the adoptive transfer of HLA-C*08:02-restricted T cells specific for KRAS G12D, which induced the regression of metastatic pancreatic ductal adenocarcinoma in a single patient [17]. Consequently, a practical pre-manufactured product within this category is a mutation-HLA library rather than a single construct.

### 3.3. Mismatch-Repair-Derived Abnormal Peptides

The third category encompasses the recurrent abnormal peptide family produced because of mismatch-repair deficiency. In individuals with Lynch syndrome and sporadic microsatellite instability (MSI), repetitive coding sequences, such as the mononucleotide tracts in TGFBR2, BAX, ACVR2A, and MSH3, are susceptible to recurrent frameshift mutations when mismatch repair mechanisms fail, leading to the generation of abnormal peptide sequences that do not present in normal proteins. Since the same coding microsatellites are prone to slipping across different patients, identical peptide classes are observed among patients with mismatch-repair-deficient tumors. This biological phenomenon renders Lynch syndrome and MSI the most credible current experimental models for cancer interception, although clinical prevention through vaccination remains unproven.

### 3.4. Hematologic Mutation-Derived Neoantigens

Hematologic examples necessitate the use of more precise terminology than is sometimes employed in the literature. CALR exon 9 insertions and deletions in myeloproliferative neoplasms induce a +1 frameshift, resulting in a novel positively charged C-terminus; the mutant C-terminal peptides are shared neoantigens among CALR-mutant patients [18]. NPM1-mutated acute myeloid leukemia involves small insertions, notably a 4-base-pair duplication in exon 12, that modify the C-terminus of the NPM1 protein and produce new peptides presented on HLA; these are recurrent mutant C-terminal neoantigens [19] and do not constitute classical MSI-style frameshift peptides. This distinction is significant because the mechanisms of recurrence differ, as do the range of peptides and HLA coverage that emerge.

### 3.5. Fusion-Junction Peptides

Fusion-junction peptides arise from chromosomal rearrangements, resulting in chimeric proteins that lack junction sequences found in normal proteins. The BCR-ABL fusion was the initial prototype; however, tyrosine kinase inhibitor therapy has largely supplanted fusion-junction vaccination as the primary treatment approach in chronic myeloid leukemia. In principle, shared targets such as EWS-FLI1 in Ewing sarcoma and specific kinase and transcription factor fusions in other malignancies are feasible; however, the heterogeneity of breakpoints and HLA restriction limits the universality of any single construct. For EWS-FLI1 specifically, the fusion structure is well defined, but natural peptide–HLA presentation of the junction tile and tumor-cell recognition by T cells have not been demonstrated; it is therefore classified here as sequence-defined but not presentation-validated.

### 3.6. Tumor-Associated Antigens (Explicitly Excluded)

Proteins that are overexpressed, cancer-testis antigens, tissue-restricted proteins, differentiation antigens, stemness markers, and oncofetal antigens may support therapeutic strategies in specific contexts; however, they do not satisfy the strict criterion of being exclusively present in cancer cells. Markers such as HER2, MUC1, hTERT, WT1, PSMA, survivin, NY-ESO-1, PRAME, mammaglobin, and alpha-lactalbumin [20,21] are each expressed in specific normal tissues or during specific developmental stages. They are incorporated into discussions of breast and gastrointestinal cancer prevention solely when the tissue-restricted nature of the target is explicitly recognized. The lack of success in vaccines targeting MAGE-A3, EGFRvIII, PSA, and MUC1 in advanced disease, as summarized in Section 2.5, reinforces this principle (Table 1).

Table 2 lists the 15 sequence-anchored constructs and the 1 conceptual placeholder across the 13 target candidates. Each KRAS allele is treated as a separate construct module within the same target class. Each HPV E6 and E7 construct is treated as a separate module within the viral oncoprotein class. The CALR exon 9 frameshift is treated as a single construct because the type 1 and type 2 mutations converge on one shared novel C-terminus. The “sixteen construct modules” total (fifteen sequence-anchored plus one conceptual placeholder) reflects construct modules, not biologically independent target classes.

## 4. Mechanism: T-Cell Education, Not Epitope Blockade

An mRNA vaccine does not physically obstruct an intracellular epitope. Most of the targets discussed are intracellular peptides presented on HLA molecules; direct antibody blockade is not the primary mechanism. The mRNA encodes one or more antigenic regions that encompass the relevant mutation, viral oncoprotein segment, frameshift sequence, insertion-altered C-terminus, or fusion junction. Following uptake by antigen-presenting cells, the mRNA is translated, and the resulting protein or peptide antigen is processed. The processed peptides are subsequently loaded onto HLA class I and class II molecules, and the peptide–HLA complexes are presented to CD8+ and CD4+ T cells. If the same peptide–HLA complex is naturally presented on a malignant cell, trained T cells can recognize and eliminate that cell [22].

This mechanism is selective only when the target peptide–HLA complex is genuinely absent from essential normal tissues. Predicted HLA binding alone does not constitute sufficient evidence of natural presentation; empirical validation using tumor HLA molecules, via immunopeptidomics or direct T-cell recognition of tumor cells, is necessary before considering a candidate a validated target. Tumors may evade this mechanism by losing antigen expression, deleting HLA alleles, down-regulating beta-2 microglobulin, suppressing interferon responses, excluding T cells, or recruiting suppressive myeloid populations. The most appropriate targets are clonal, early, and functionally significant. Strategies involving multiple epitopes and combinations with checkpoint blockade or other forms of immune modulation may be required.

Modified nucleoside mRNA chemistry, initially demonstrated by showing that pseudouridine incorporation decreases innate immune recognition and improves translation [23,24], and reviewed for vaccine applications [22], enables large-scale manufacturing of mRNA. The phase 3 trials of the BNT162b2 and mRNA-1273 SARS-CoV-2 vaccines demonstrated that nucleoside-modified mRNA can be produced, distributed, and administered at scale to broad populations with an acceptable safety profile [25,26]. Post-authorization experience has expanded the safety database substantially, but infectious-disease platform experience does not establish oncology-specific safety, dosing, or efficacy. These findings confirm the feasibility of platform manufacturing and short-term safety; however, they do not establish the safety, dosing, reactogenicity, or efficacy profiles of multi-antigen tumor-specific cancer vaccines, which differ fundamentally in target biology, dosing regimens, immune environments, and clinical populations. Extrapolation from infectious-disease mRNA vaccines to tumor-specific mRNA vaccines is limited to platform feasibility, necessitating the re-establishment of distinct safety, tolerability, dosing, and clinical efficacy for each oncology indication. This consideration applies to all programs discussed in Section 10 and is not separately reiterated there.

**Figure 2:**
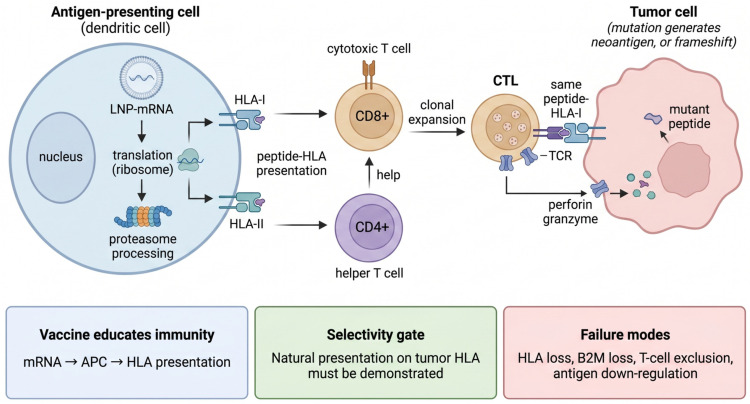
A mechanistic schematic illustrating the uptake of mRNA by antigen-presenting cells, subsequent antigen translation, intracellular processing, and the presentation of peptide–HLA class I and class II complexes. The diagram also depicts T cell recognition of tumor cells, which naturally present identical peptide–HLA complexes. Although the schematic depicts a single tumor cell, the target peptide–HLA complex represents any of the target classes considered in this manuscript: a viral oncoprotein, a driver mutation, a frameshift-derived peptide, an insertion-altered C-terminus, or a fusion-junction peptide. The purpose of the vaccine is to educate the immune system, rather than to directly inhibit intracellular epitopes. Created in BioRender. Niazi, S. (2026) https://BioRender.com/u6t8q34.

A fundamental safety principle emerges from the mechanism: genetically stable oncogenic proteins should not be encoded in forms that retain their transformative capabilities. For HPV E6 and E7, constructs should be engineered as non-transforming antigenic modules, for instance by mutating residues that bind p53 and retinoblastoma, while preserving T-cell epitopes. The representative HPV constructs shown in the sequence-design section (Section 17) are attenuation concepts, not experimentally validated non-transforming vaccine inserts. Regarding driver mutations, the construct should encompass sufficient flanking sequences to support natural antigen processing, thereby avoiding unnecessary expression of functional proteins. These are guiding principles rather than strict directives, and any specific construct must undergo product-level regulatory review (Figure 2).

## 5. BRCA-Associated Breast Cancer: A Corrective Case Study

**Figure 3:**
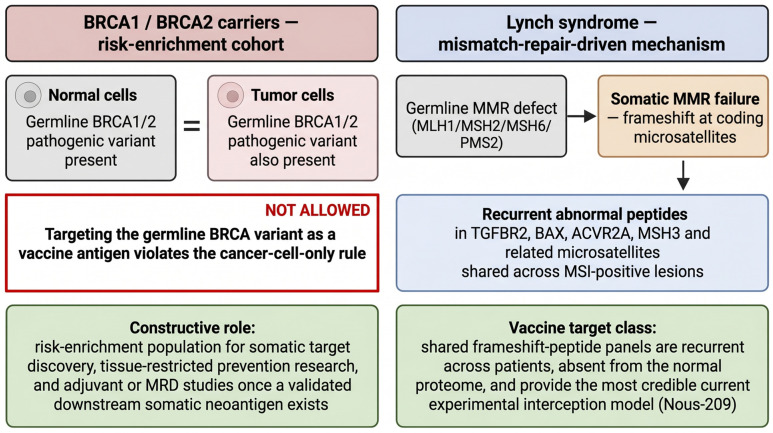
Comparison between the BRCA risk cohort and the Lynch syndrome mechanism. Pathogenic variants in BRCA1 and BRCA2 delineate a high-risk population but do not inherently specify a tumor-only antigen. Lynch syndrome, characterized by defects in mismatch-repair genes, presents a predictable mechanism whereby somatic frameshift mutations recur in both premalignant and malignant tissues following mismatch-repair failure. Consequently, Lynch syndrome offers a more direct framework for research into shared-antigen interception. Created in BioRender. Niazi, S. (2026) https://BioRender.com/u6t8q34.

BRCA-associated breast cancer is the most important case study for the prevention argument because of its clinical familiarity, emotional resonance, and the scientific tendency to exaggerate. BRCA1 and BRCA2 are DNA repair genes; inherited pathogenic variants in these genes raise the lifetime risk of breast, ovarian, pancreatic, prostate, and other cancers [27,28]. An inherited pathogenic BRCA variant is a germline marker present in normal, precancerous, and cancerous cells. Immune targeting of a germline BRCA variant, therefore, does not meet the cancer-cell-specific criterion (Figure 3). Two distinct uses of BRCA must be kept separate and not conflated. The first is risk-enrichment for target discovery: BRCA1 and BRCA2 carriers constitute a population in which somatic tumor-specific targets can be sought efficiently, and this use is legitimate. The second is vaccine-target validity: the question of whether any specific antigen meets the strict cancer-cell-only rule. A germline BRCA variant satisfies the first use but fails the second. To be unambiguous for non-specialist readers, the inherited BRCA1 or BRCA2 variant is itself a germline risk marker and is not a vaccine antigen; only validated downstream somatic neoepitopes, junctions, or splice events arising within BRCA-associated tumors, demonstrated to be naturally presented and absent from essential normal tissue, could qualify as vaccine targets, and none has yet been validated.

There is no validated evidence that all carriers of BRCA1 or BRCA2 mutations, or all BRCA-associated breast cancers, possess a universal tumor-only epitope suitable for a non-personalized vaccine. Small computational analyses have identified candidate shared neoantigens within subsets of BRCA1-related breast cancer using public databases [29]; these candidates remain exploratory and have not been validated by natural presentation, T-cell recognition, or normal-tissue safety data. They do not constitute a clinically usable vaccine target class and should not be presented as a solution for BRCA prevention.

The constructive utilization of BRCA within this framework serves as a population enriched for risk, intended for target discovery. An exhaustive discovery program would scrutinize premalignant tissue, samples obtained via prophylactic mastectomy or salpingo-oophorectomy, ductal carcinoma in situ, high-grade serous ovarian precursor lesions, and clinically manifest tumors from BRCA-defined subgroups. It would identify recurrent somatic mutations, junctions, or splice events, verify natural peptide–HLA presentation using immunopeptidomics or tumor-recognition assays, and evaluate safety in normal tissues before proposing a shared mRNA library. BRCA1-associated breast cancers are enriched for a triple-negative phenotype and differ from BRCA2-associated tumors in mutational pattern and clinical progression; therefore, a universal BRCA-based vaccine target is biologically improbable. Any future shared neoepitope library would more plausibly be subgroup-specific (for example, BRCA1-associated triple-negative breast cancer with a particular recurrent somatic mutation and HLA restriction).

Discussions regarding concepts for breast cancer prevention vaccines may still be ongoing; however, it is imperative that the terminology employed remains precise. Alpha-lactalbumin, a tissue-restricted self-antigen predominantly expressed during lactation, has been examined as a preventive target in cases of triple-negative breast cancer and among women contemplating prophylactic mastectomy [21,30]. Its clinical utility may be pertinent after childbearing or in patients already considering risk-reduction surgeries. It should be noted that alpha-lactalbumin is not a neoepitope exclusive to tumor cells. The INO-5401 vaccine, administered via intramuscular electroporation in carriers of BRCA1 and BRCA2 mutations, encodes tumor-associated antigens such as hTERT, PSMA, and WT1 in the form of a plasmid [31], and is not an mRNA construct; moreover, its targets are tumor-associated but not limited to cancer cells. The antigens MUC1, HER2, mammaglobin, hTERT, WT1, and survivin are explicitly omitted from the central shared tumor-specific library as delineated within this manuscript. These antigens are classified as tissue-restricted or tumor-associated but do not meet the strict definition of tumor-only antigens and may still retain clinical relevance in carefully defined contexts where safety considerations are acknowledged (see Table 3).

## 6. Prevention and Interception

The phrase “cancer prevention vaccine” must be understood as having two distinct meanings. The first pertains to the prevention of infections that have the potential to cause cancer, which has been substantiated at the population level using HPV vaccination [32,33,34] and hepatitis B vaccination [35], as summarized in Section 2.1. Evidence indicates that these vaccines prevent infection or chronic viral diseases; however, they do not induce T cell responses capable of eliminating premalignant human cells that already possess a somatic neoepitope. The second meaning involves the immunologic interception of premalignant or newly malignant cells, a concept that remains within the experimental phase.

Lynch syndrome is currently the most defensible experimental model for interception. The inherited condition does not provide a vaccine target; it predisposes to mismatch-repair failures that produce recurrent abnormal frameshift-derived peptide classes in both premalignant and malignant tissue. The Nous-209 program advances this concept through a heterologous prime-boost approach (great-ape adenovirus GAd20 as the prime and modified vaccinia Ankara as the boost), delivering 209 shared frameshift peptide neoantigens. In a phase 1b/2 single-arm trial of 45 Lynch syndrome carriers (37 evaluable for immunogenicity), the vaccine was well tolerated, with no serious treatment-related adverse events, and elicited neoantigen-specific CD8+ and CD4+ T-cell responses in all 37 evaluable participants, with responses persisting for up to one year in 85% [36]. More than 100 immunogenic frameshift peptides were identified and validated across independent datasets of Lynch-associated MSI precancers and cancers [36].

The study offers biological proof of concept. However, a reduction in cancer incidence among Lynch syndrome carriers has not yet been demonstrated. Confirmatory evidence necessitates larger and more extended randomized trials with endpoints such as adenoma formation, advanced neoplasia, or cancer incidence. The example is referenced here to support the target-class rationale, rather than as evidence of a clinical outcome of an mRNA platform, since the published platform utilizes a viral vector rather than an mRNA platform.

Primary prevention studies necessitate considerable time investment. A trial intended to demonstrate a reduction in cancer incidence among high-risk, yet cancer-free participants typically requires several years and extensive cohorts. More expedited mechanistic evaluations can employ immune responses, biopsy-based target recognition, adenoma burden, circulating tumor DNA, molecular residual disease, or recurrence-free survival following the removal of an early cancer. Consequently, the manuscript distinguishes between proof of immunogenicity, proof of target engagement, proof of interception, and proof of cancer incidence reduction; these concepts are not interchangeable (see Table 4).

## 7. HPV-Driven Cancers: The Lead Therapeutic Archetype

HPV-driven cancers represent the most well-defined category of shared tumor-specific antigens appropriate for non-personalized therapeutic vaccination. HPV16 and HPV18 E6 and E7 are viral oncoproteins, distinct from normal human proteins, and they maintain the malignant phenotype in HPV-positive cancers; the loss of antigens through selective processes is less feasible than for dispensable passenger antigens. These cancers encompass cervical, anal, vulvar, vaginal, and penile malignancies, as well as a significant portion of oropharyngeal head and neck squamous cell carcinoma [33]. The population-level prevention data discussed in Section 2.1 [37,38,39] do not directly translate to therapeutic vaccination but demonstrate that immunization against these viral antigens can be carried out safely on a large scale.

A therapeutic E6/E7 mRNA vaccine does not substitute for prophylactic HPV vaccination [32,34]. Prophylactic vaccination prevents infection before cellular transformation; therapeutic vaccination targets cells that have already begun expressing viral oncoproteins. The two product categories are complementary rather than interchangeable.

Within the therapeutic mRNA category, BNT113 is the most publicly described current investigational example. The publicly available description characterizes BNT113 as a vaccine encoding HPV16 E6 and E7, designed to induce CD8+ and CD4+ T-cell responses against viral oncoprotein-derived peptide–HLA complexes following antigen processing and presentation [40]. In January 2026, BioNTech reported that the U.S. Food and Drug Administration granted Fast Track designation to BNT113 in combination with pembrolizumab for HPV16-positive, PD-L1-expressing recurrent or metastatic head and neck squamous cell carcinoma [41], and the sponsor describes the ongoing AHEAD-MERIT trial (BNT113-01) of BNT113 plus pembrolizumab versus pembrolizumab alone in this population [42]. BNT113 should be cited only as an investigational program with Fast Track regulatory status and an ongoing trial whose results are pending; it should not be cited as evidence of established benefit, pivotal success, or likely approval. Fast Track designation is a regulatory facilitation pathway and is not a measure of clinical efficacy. Clinical efficacy remains under evaluation, and any final position will depend on results not yet in the public record.

Non-mRNA HPV therapeutic vaccines reinforce the same target logic and are valuable comparators. The HPV16 synthetic long-peptide vaccine ISA101, in combination with nivolumab, demonstrated clinical activity in patients with incurable HPV16-positive cancers in a phase 2 trial [43]. PDS0101, when combined with pembrolizumab, is currently under evaluation in a phase 3 trial targeting HPV16-positive recurrent or metastatic head and neck squamous cell carcinoma (HNSCC) [44]. The DNA-based therapeutic vaccine VB10.16, combined with atezolizumab, has reported safety and clinically significant efficacy signals in HPV16-positive advanced cervical cancer [45]. Although these programs are platform-specific and not interchangeable, they converge upon the same fundamental target strategy: vaccination against fixed viral oncoproteins combined with checkpoint blockade.

A practical product family within the HPV class will probably require HPV16 and HPV18 modules, and potentially additional high-risk HPV-type modules, with clinical development structured by tumor site, PD-L1 status, disease burden, and the use of combination therapies such as checkpoint blockade or other agents. The HPV class is included here as the primary pre-manufactured therapeutic archetype because it meets the strict target criteria, not because of established clinical efficacy.

## 8. KRAS-Mutant Cancers and Pancreatic Ductal Adenocarcinoma

KRAS constitutes the largest nonviral shared driver family within a pre-manufactured vaccine library, as recurrent hotspot mutations at codons 12 and 13 are observed across various cancers and are integral to malignant biology. The vaccine targets mutation-specific peptides (G12D, G12V, G12R, G12C, or G13D) presented by compatible HLA molecules, rather than wild-type KRAS. While this criterion restricts population coverage, it enhances biological specificity.

Pancreatic ductal adenocarcinoma (PDAC) is the most compelling early solid-tumor model because approximately 90% of cases harbor KRAS mutations [46]. Recurrence following surgical resection is prevalent, developing within two years in the majority of resected patients [47], and the adjuvant or molecular residual disease contexts offer practical clinical endpoints within manageable timeframes [48]. The identity of KRAS alleles and the co-mutational landscape are correlated with clinical outcomes [49,50], thereby supporting the rationale for mutation-specific targeting and highlighting that not all KRAS-mutant PDACs are clinically equivalent.

The evidence base for KRAS-directed vaccination must be discussed by platform, because the available clinical data come from different approaches. The personalized RNA neoantigen vaccine autogene cevumeran, administered with anti-PD-L1 therapy and modified FOLFIRINOX after surgery for resectable PDAC, elicited neoantigen-specific T cells in approximately 50% of enrolled patients in a phase 1 trial, and vaccine-induced T-cell responders showed delayed recurrence compared with non-responders [51]. At a 3.2-year follow-up, vaccine-induced CD8+ T cells remained durable, with an estimated half-life consistent with long-term memory, and the recurrence-free survival difference between responders and non-responders persisted [52]. These findings support the general hypothesis that RNA neoantigen vaccination can induce mutation-specific T-cell responses in PDAC. The approach is personalized in design and does not establish the efficacy of a shared KRAS vaccine.

The AMPLIFY-201 program evaluates a pre-manufactured shared-KRAS approach utilizing the lymph-node-targeting amphiphile peptide vaccine ELI-002 2P. ELI-002 2P comprises amphiphile-modified mutant KRAS G12D and G12R peptides in conjunction with an amphiphile-CpG adjuvant (CpG-7909). In a phase I clinical trial involving 25 patients (20 with pancreatic ductal adenocarcinoma (PDAC) and 5 with colorectal cancer) exhibiting minimal residual mutant-KRAS disease following standard locoregional therapy, the vaccine elicited mutant-KRAS-specific T-cell responses in 84% of the cohort [53]. Both CD4+ and CD8+ T-cell subsets were induced in 71% of evaluable patients, with evidence of antigen spreading to additional tumor antigens in 67%. The final analysis of phase I data, with a median follow-up of 19.7 months, reported a hazard ratio of 0.12 for radiographic relapse-free survival (*p* = 0.0002) among patients with T-cell responses exceeding a 9.17-fold increase over baseline [54]. In this subgroup, median relapse-free survival was not reached, compared to 3.02 months in the below-threshold subgroup. A parallel trend was observed for overall survival (hazard ratio 0.23, *p* = 0.0099), with median overall survival not reached versus 15.98 months [54]. The results of AMPLIFY-201 support a shared-KRAS pre-manufactured strategy and highlight the significance of T-cell response thresholds as biomarkers indicative of therapeutic benefit. ELI-002 is a peptide-amphiphile rather than mRNA; consequently, it should not be cited as direct evidence supporting an mRNA-based KRAS therapeutic.

V941 (mRNA-5671) is the most prominently documented mRNA-derived KRAS-targeted vaccine concept available in the public domain. The NCI Drug Dictionary describes it as targeting four KRAS hotspot mutations (G12D, G12V, G13D, and G12C) and relying on antigen-presenting cell uptake, translation, and presentation via both class I and class II HLA molecules [55]. The program should be regarded as a historical example of a publicly available mRNA-KRAS concept rather than evidence of current clinical activity; NCI describes the construct, while subsequent trial and sponsor or trade-status sources indicate the program is no longer an active clinical pipeline asset. The phase 1 study (ClinicalTrials.gov NCT03948763) evaluated mRNA-5671/V941 as monotherapy and with pembrolizumab in KRAS-mutant non-small-cell lung cancer, colorectal cancer, and pancreatic adenocarcinoma [56], and trade reporting indicates that the co-development partner discontinued the program after closing enrollment in that phase 1 trial [57]. V941 is included here as an illustrative historical example of an mRNA-platform shared-KRAS concept, not as an active component of the current clinical pipeline.

The KRAS literature advocates a development paradigm in which a non-personalized mRNA product functions as a mutation-HLA library rather than a single construct. It is imperative that each module demonstrate HLA binding, natural tumor presentation, mutation-specific T-cell recognition, absence of cross-reactivity with wild-type KRAS, and sufficient population coverage as determined by HLA-frequency analysis. The adjuvant or minimal residual disease (MRD) setting in pancreatic ductal adenocarcinoma (PDAC) represents the most practical initial clinical context for demonstrating efficacy. Furthermore, the distribution of KRAS alleles across various cancer sites, such as PDAC, colorectal cancer, and lung adenocarcinoma, dictates the scope of applicability for any single library [58].

## 9. Other Priority Targets

This section discusses the remaining shared tumor-specific target classes in turn. Subsection numbering treats each topic at the same level for parity, including the platform-benchmark reference to personalized mRNA neoantigen vaccination.

### 9.1. IDH1 R132H Glioma

IDH1 R132H glioma represents a prominent example of a shared clonal mutation-derived neoepitope within a specific subgroup. The mutation yields a class II-restricted peptide that elicits mutation-specific CD4+ T-cell responses. Preclinical studies demonstrated antitumor efficacy in animal models [59], and the initial human NOA-16 trial in newly diagnosed IDH1 R132H-mutant astrocytoma reported safety and high frequencies of mutation-specific immune responses. The three-year progression-free and overall survival rates were comparable to historical data, although the trial was not randomized [60]. Current evidence supports a mechanistic basis rather than a definitive survival advantage.

### 9.2. H3 K27M Diffuse Midline Glioma

H3 K27M diffuse midline glioma represents a specialized focus area. An early-phase peptide vaccine targeting H3 K27M has demonstrated safety and has elicited mutation-specific T-cell responses, including a case of durable complete response following pseudoprogression [61]. The disease is rare, aggressive, and clinically constrained by neurologic considerations, and the trial sample size is limited. This example illustrates that a shared histone-mutation vaccine can induce mutation-specific immunity; however, it does not establish efficacy. SurVaxM, a peptide vaccine targeting the tumor-associated antigen survivin, has reported phase 2 data in newly diagnosed glioblastoma [62]; survivin is not exclusively a cancer neoepitope, and the program is presented here as a tumor-associated comparator rather than a strictly tumor-specific entity.

### 9.3. NPM1-Mutated Acute Myeloid Leukemia

NPM1-mutated acute myeloid leukemia holds significance owing to the insertion-modified C-terminus, which generates novel HLA-presented peptides that are directly identified on AML cells through immunopeptidomics [19]. The mutation is widespread, and the disease can be monitored with high sensitivity utilizing molecular residual disease assays, allowing for serial sampling. Within this context, vaccine evaluation would most appropriately occur during molecular remission, characterized by a low tumor burden and the ability to monitor molecular relapse.

### 9.4. CALR-Mutated Myeloproliferative Neoplasms

Mutations in CALR exon 9 observed in essential thrombocythemia and primary myelofibrosis give rise to a distinctive shared frameshift neoantigen at the C-terminus [18]. A peptide vaccine targeting mutant CALR has been investigated in patients with CALR-mutant myeloproliferative neoplasms; preclinical studies demonstrated that these tumors can alter MHC class I expression, and that such alterations may be mitigated by an optimized peptide vaccine [63]. Like NPM1, pertinent clinical endpoints encompass immune response, mutant allele burden, symptom burden, and indicators of disease progression rather than immediate response rates.

### 9.5. Merkel Cell Polyomavirus and Merkel Cell Carcinoma

Merkel cell polyomavirus-positive Merkel cell carcinoma constitutes the primary viral oncoprotein context outside the human papillomavirus (HPV) category. The integrated truncated MCPyV large T antigen promotes cellular proliferation in virus-positive cases, while the small T antigen also contributes to oncogenic transformation. Both antigens are foreign to the host, frequently observed in virus-positive disease, and are conceptually analogous to HPV E6 and E7 [16]. The development of therapeutic vaccines is less advanced than for HPV, and a combination strategy with anti-PD-1 therapy is essential, given that PD-1 blockade is already a standard treatment for advanced Merkel cell carcinoma. The MCPyV capsid antigen VP1 does not serve as the primary tumor-maintaining antigen in established Merkel cell carcinoma; although VP1-directed research may have prophylactic implications, it should not be regarded as the central evidence for a shared tumor-specific Merkel cell carcinoma vaccine. As outlined in the sequence-design section (Section 17), MCPyV is presented in this manuscript at the design-specification level solely and is not a sequence-anchored construct.

### 9.6. Epstein–Barr Virus-Associated Cancers

Epstein–Barr virus (EBV)-associated malignancies are considered promising targets owing to the foreign nature of viral antigens; however, the biological complexity exceeds that observed in HPV-positive cancers. Variations in EBV latency programs are evident across various malignancies, including nasopharyngeal carcinoma, EBV-positive gastric carcinoma, Hodgkin lymphoma, NK/T-cell lymphoma, and post-transplant lymphoproliferative disease. Consequently, a vaccine target effective in one EBV-associated cancer may not be applicable to another [64]. An early-stage EBV-directed mRNA approach is exemplified by WGc-043; in 2024, the U.S. Food and Drug Administration (FDA) cleared (allowed) the Investigational New Drug (IND) application for this candidate, permitting clinical investigation. WGc-043 is not FDA-approved as a therapeutic product, and the IND clearance does not constitute a product approval or any claim regarding clinical efficacy. Currently, no efficacy data for WGc-043 are publicly available [65,66]. The immediate prospects are focused on diseases with consistent and quantifiable EBV antigen expression, such as nasopharyngeal carcinoma and certain EBV-positive lymphomas. EBV latent antigen expression is not uniform across the spectrum of EBV-associated disorders; therefore, each potential indication necessitates confirmation of the latency pattern (I, II, III, or lytic) and verification that the specific T-cell target peptides are naturally presented by human leukocyte antigen (HLA) molecules on the pertinent tumor cells. Accordingly, the framework does not consider EBV as a singular, homogeneous viral oncoprotein class. Success in this domain will depend on latency-informed antigen selection, biomarker confirmation of EBV positivity, indication-specific verification of antigen expression, and combination strategies to overcome tumor immune evasion.

### 9.7. Platform Benchmark: Personalized mRNA Neoantigen Vaccination

**Figure 4:**
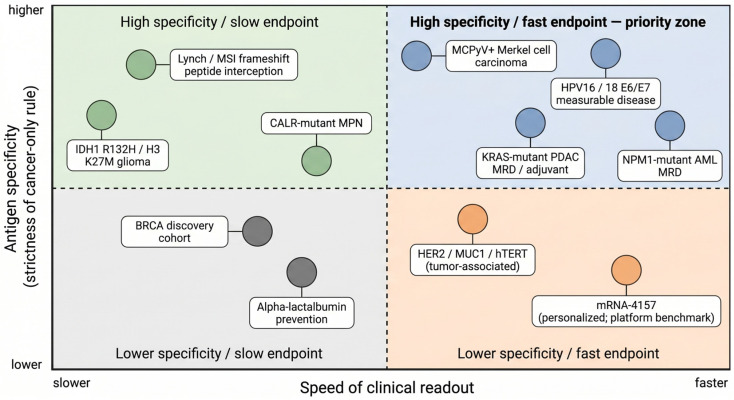
Qualitative Priority Matrix. The conceptual matrix contrasts antigen specificity (vertical axis) with the practical speed of clinical readout (horizontal axis). Positions are qualitative and do not imply a formal scoring model or numerical effect sizes; HPV-positive measurable disease, NPM1 AML MRD, and PDAC/KRAS MRD occupy positions conducive to rapid readout, whereas Lynch interception and BRCA discovery are situated in slower-readout positions. mRNA-4157/V940 appears only as a personalized mRNA platform benchmark and is not a shared or pre-manufactured product; its position reflects platform-level feasibility, not evidence for shared fixed-antigen vaccines. The matrix reflects authorial judgment and is not an evidence-based ranking or a prediction of clinical success. Created in BioRender. Niazi, S. (2026) https://BioRender.com/u6t8q34.

The personalized mRNA neoantigen vaccine mRNA-4157 (V940), in combination with pembrolizumab, has demonstrated an extension in recurrence-free survival compared with pembrolizumab alone in patients with resected high-risk melanoma in the KEYNOTE-942 phase 2b trial [67]. This constitutes substantial platform-level evidence indicating that mRNA neoantigen vaccination can be clinically efficacious when used alongside checkpoint blockade in an adjuvant setting. mRNA-4157 is a bespoke product rather than a shared-antigen formulation. The results of the KEYNOTE-942 trial, which were obtained through a patient-specific antigen design, do not imply that shared, fixed-antigen mRNA constructs will be efficacious in other tumor types. Each shared-target indication discussed in this manuscript necessitates its own clinical assessment, and the findings of KEYNOTE-942 should not be construed as evidence supporting shared pre-manufactured products (Table 5) (Figure 4).

## 10. The mRNA Platform: Capabilities and Current Pipeline in Oncology

mRNA is a flexible platform for the development of cancer vaccines due to its ability to encode virtually any peptide sequence, support multi-antigen constructs, and utilize a largely consistent manufacturing framework across various antigen sequences; its clinical performance nonetheless depends on antigen biology, delivery, immune context, dosing, and indication-specific validation. Module-level updates are more straightforward compared to many alternative vaccine technologies, providing an advantage for a library of shared mutations, frameshifts, or viral-antigen vaccines. The platform also benefits from over a decade of research on nucleoside modifications [23,24] and extensive phase 3 validation in infectious diseases [25,26]. The mechanism, capabilities, and the explicit limitation that the feasibility in infectious diseases does not imply demonstrated safety, dosing, or efficacy in oncology are discussed in Section 4 and are not reiterated here.

Beyond BNT113 in HPV16-positive HNSCC (Section 7), several non-personalized mRNA cancer vaccines are in clinical or near-clinical development. BNT116 has entered its first-in-human phase 1 evaluation (LuCa-MERIT-1) for non-small-cell lung cancer, administered as monotherapy and in combination cohorts [68], with a randomized phase 2 trial (EMPOWERVAX Lung 1, NCT05557591) currently underway. EMPOWERVAX Lung 1, conducted collaboratively by BioNTech and Regeneron, compares BNT116 plus cemiplimab with cemiplimab alone in patients with advanced non-small-cell lung cancer expressing PD-L1 at or above 50%. This ongoing phase 2 study has not yet reported efficacy data [69]. BNT111, a melanoma FixVac candidate encoding shared melanoma-associated antigens, has reported phase 2 topline data with cemiplimab in a sponsor disclosure [70], and subsequent trade reports indicate that the sponsor does not intend to pursue further trials in the late-stage refractory melanoma setting [71]. The mixed signals surrounding BNT111 suggest it should not be regarded as a definitive model of shared non-personalized mRNA success but as an illustration of platform feasibility amid ongoing commercial uncertainty. Moderna’s pipeline includes mRNA-4359, an investigational cancer antigen therapy that encodes epitopes of PD-L1 and IDO1 [72,73]. mRNA-4359 has received FDA Fast Track designation in combination with pembrolizumab for checkpoint-inhibitor-refractory unresectable or metastatic PD-L1-positive melanoma (tumor proportion score > 1%), and Phase 2 dose-expansion cohort data within the Phase 1/2 study (NCT05533697), evaluating mRNA-4359 plus pembrolizumab as first-line therapy in locally advanced or metastatic melanoma, were presented at AACR 2026 [74]. The Fast Track designation and the first-line cohort refer to different patient populations and should not be conflated; neither provides conclusive evidence of clinical benefit. Everest Medicines announced in November 2025 that the first patient had been dosed in a global phase 1 trial of EVM14, an mRNA vaccine targeting tumor-associated antigens for squamous cell carcinomas; efficacy data are currently pending [75]. CureVac received FDA Investigational New Drug authorization for CVHNLC, an mRNA-based precision immunotherapy targeting squamous non-small-cell lung cancer; this authorization permits clinical investigation but does not imply therapeutic efficacy [76]. CureVac also reported in a quarterly business update that phase 1 part B of CVGBM, an mRNA glioblastoma vaccine encoding tumor-associated epitopes, was fully enrolled; however, enrollment status alone does not establish efficacy [77]. For all these programs, status updates are derived from sponsor or regulatory communications, and none demonstrate proven clinical benefit. Although the pipeline is expanding, it remains predominantly investigational, with individual programs susceptible to delays, modifications, or discontinuation.

Platform flexibility does not resolve the issue of antigens. A vaccine encoding a meticulously designed target in silico may still fail if the target is not naturally presented on tumor HLA molecules, if the tumor diminishes HLA expression under immune pressure, or if the tumor microenvironment obstructs effector T-cell infiltration. Manufacturing output, in and of itself, cannot confirm that a KRAS, BRCA-associated, or frameshift target is tumor-specific and protective. Many of the candidates encode tumor-associated antigens rather than strictly cancer-specific epitopes; the considerations in Section 3.6 and Section 14 are directly applicable.

The KEYNOTE-942 result with mRNA-4157 plus pembrolizumab [67] in resected melanoma is an important indication that the mRNA platform can be clinically active in oncology, but that result was achieved in a personalized rather than shared-antigen configuration and in combination with checkpoint blockade.

To make the evidence tier for each current program explicit, Table 6 lists the 2024–2026 pipeline references, including their target, disease, trial identifier, source type, latest verified status, and what each source does not establish (Table 6).

## 11. Product Concept Without Executable Detail

This manuscript delineates non-operational product logic. The sequence-design section (Section 17) supplies representative, human-codon-compatible open reading frame (ORF) sequences for all fifteen sequence-anchored candidates and includes a design-specification-only entry for MCPyV. It excludes lipid-nanoparticle formulations, dosing schedules, comprehensive mRNA transcript layouts (including 5′ UTR, cap, and poly-A tail length), and manufacturing parameters. The provided ORFs demonstrate the encoded antigenic region, the position of mutations or safety substitutions, and the delineation between encoded T-cell epitopes and the exclusion of oncogenic protein function. It is important to note that these sequences are not validated for clinical application. Product development must occur within authorized laboratories and regulated clinical environments; furthermore, any specific construct necessitates approval from institutional, regulatory, biosafety, and ethics authorities. Such constraints are particularly pertinent for viral oncoprotein targets and preventive strategies aimed at high-risk populations, where public communication must refrain from suggesting that a conceptual vaccine is ready for preventive deployment.

The initial step in product-concept development involves delineating coverage, including the identification of relevant viral types and tumor sites associated with HPV- and EBV-driven malignancies. Additionally, it entails determining mutation alleles and HLA compatibility for KRAS, IDH1, and H3 K27M. The KRAS module is incorporated as a sequence-anchored reference design rather than as an active mRNA-vaccine pipeline asset, given that the sole previously named shared mRNA-5671/V941 clinical program has been discontinued, as detailed in Section 8 and Table 5. Furthermore, the process involves establishing inherited syndromes alongside validated frameshift panels for Lynch syndrome and MSI-related biology and specifying antigen classes and disease states for NPM1 and CALR within hematologic contexts. The second step concerns target discovery, in which candidate regions are prioritized based on tumor-only status, recurrence, clonal significance, natural presentation, and immunogenic potential. For mutation targets, the mutant residue must be flanked by adequate sequences to support natural antigen processing while avoiding the formation of an intact oncogenic protein. For viral oncogenes, antigenic regions should be engineered to be non-transforming while retaining the epitope content necessary for T-cell recognition. For frameshift peptide panels, the composition should reflect HLA polymorphism and the recurrence frequency of each frameshift within the relevant population.

Evidence layers should be reported separately rather than collapsed. Predicted HLA binding, empirically detected peptide–HLA presentation on tumor cells, vaccine-induced T-cell responses, direct tumor-cell recognition by induced T cells, and clinical benefit are distinct levels of evidence, and prediction alone is not validation. For prevention and other low-risk settings, normal-tissue safety and cross-reactivity testing deserve at least as much emphasis as manufacturability.

## 12. Validation Gates and Clinical Endpoints

A comprehensive program incorporates explicit go/no-go decision points. The initial criterion is tumor specificity, which involves a defensible absence of the target in essential normal tissues at the peptide–HLA level relevant to the immune response. Normal-tissue transcriptomic and proteomic data are valuable, and the combination of tumor and normal-tissue immunopeptidomics with cross-reactivity testing in T-cell assays is more directly relevant for the development of T-cell vaccines. The second criterion involves population recurrence and coverage, defined by mutation allele, viral type, cancer site, HLA allele frequency, and disease stage. Coverage claims must consider HLA distribution differences across ancestral populations, as allele frequencies vary significantly; hence, global equity of a vaccine library depends on this distribution. The OSE2101/Tedopi program [15] exemplifies HLA-restricted design as a precision strategy and highlights the trade-offs associated with access.

The third gate pertains to natural presentation. While HLA-binding prediction can significantly narrow the pool of candidates, empirical validation of naturally processed antigens presented by tumor cells remains essential. Predicted epitopes should not be regarded as validated targets without further evidence. The fourth gate evaluates clonality and functional importance; clonal, early, and functionally relevant targets are less likely to be lost to selection pressures than subclonal passenger antigens. The fifth gate concerns safety considerations, including the risks of recognition of normal tissues, autoimmunity, cytokine profiles, and organ-specific toxicity, particularly when indications involve significant neurologic or hematologic risks. The sixth gate emphasizes clinical interpretability; assessments should not be limited solely to metastatic response rates, nor should measurable metastatic disease be evaluated exclusively through decade-long cancer-incidence endpoints. The manuscript consistently underscores that immune responses, biomarker variations, adenoma modifications, clearance of circulating tumor DNA (ctDNA), and reductions in minimal residual disease (MRD) serve as intermediate mechanistic indicators rather than definitive evidence of long-term cancer prevention or survival benefits (see Table 7).

The tumor-specificity gate is central to this framework, so the supporting evidence is set out target by target in Table 8, which records normal-tissue transcript and protein expression, normal immunopeptidome evidence, cross-reactivity risk, available human safety data, and the resulting strict-rule classification. Predicted HLA binding alone is insufficient; the table reflects that absence of the relevant peptide–HLA complex on essential normal tissues, demonstrated as far as current methods allow, is the operative requirement.

Population recurrence and coverage depend on HLA restriction, which varies across ancestral populations. Table 9 presents the HLA dimension as an auditable construct by construct, listing known or expected HLA restriction, expected population coverage, the associated equity consideration, and the current validation status, so that the term HLA-matched library is grounded rather than assumed.

To make the evidence basis of each target family explicit and to keep distinct endpoints from being read as interchangeable, Table 9a maps every target family to its current evidence tier, the kind of supporting evidence on which that tier rests, the preferred early endpoint for the disease setting in which the family is most likely to be tested first, and the principal caveat that bounds interpretation. The evidence tiers follow the two-tier scheme defined in the Conclusion: proof-of-mechanism denotes immunogenicity, immunopeptidomic detection, single-arm early-phase data, or notable single-patient reports, whereas proof-of-benefit denotes randomized controlled data with clinical endpoints. None of the shared, pre-manufactured classes discussed here currently reaches the proof-of-benefit tier; the personalized mRNA neoantigen platform is listed separately because its randomized adjuvant melanoma data sit at a higher tier but do not transfer to shared fixed-antigen products. Immune response, ctDNA change, MRD clearance, and recurrence-free survival are not interchangeable, and the endpoint column states which readout is most appropriate for each setting.

Because the library is proposed as HLA-matched, the practical reach of each construct depends on the frequency of its restricting alleles across the major ancestry groups it would serve. Table 9b makes this quantitative by listing, for the constructs with a defined or reported restricting allele, approximate allele or phenotype frequencies across broad ancestry groupings, together with the resulting coverage implication. Frequencies are taken from large allele-frequency reference resources (the Allele Frequency Net Database [78] and published worldwide and regional HLA-frequency surveys [79,80,81]) and are given as representative point estimates or narrow ranges from named reference populations rather than exact figures for any single cohort; they vary by sub-population and reference dataset, and class II restriction for several mutation-derived tiles broadens coverage in ways not fully captured by single class I allele frequencies. The table is intended to show where coverage gaps and equity considerations arise, and which targets are supported by which alleles, so that the term HLA-matched library is grounded in explicit numbers rather than asserted.

## 13. Clinical Development Models by Target Class

Not all targets follow the same clinical trajectory. The biology of the target dictates the trial design, not the platform. For viral oncoprotein targets, the most effective initial setting is the treatment of antigen-positive measurable disease or adjuvant therapy after locoregional treatment, often combined with checkpoint blockade. Principal contexts include HPV16-positive recurrent or metastatic head and neck cancer, as well as HPV-driven cervical and anal cancers. For driver mutation targets such as mutant KRAS, the most useful initial setting is high-risk adjuvant therapy or minimal residual disease, where mutation status, MRD assays, and clinical recurrence serve as measurable endpoints within practical timeframes.

Regarding Lynch and MSI frameshift peptide vaccines, the primary emphasis is on prevention and early intervention among Lynch syndrome carriers and selected MSI-positive populations. Initial trials can demonstrate safety and immunogenicity; however, definitive prevention necessitates a reduction in advanced adenoma and cancer incidence or a delay in cancer onset. Such outcomes require extended assessment periods, and the program should not equate immune response with established cancer prevention. For hematologic targets such as NPM1 and CALR, development can include molecular residual disease monitoring and serial sampling. In acute myeloid leukemia, the pertinent question is whether vaccination after remission diminishes MRD or postpones relapse; in CALR-mutant myeloproliferative neoplasms, the focus is on whether vaccination reduces mutant allele burden, alters disease progression, or enhances long-term outcomes without unacceptable inflammatory toxicity.

In the context of brain tumor targets, neuro-oncology constraints significantly influence trial design. Factors such as blood–brain barrier penetration, steroid administration, neurological safety, pseudoprogression observed on imaging, and limited eligible populations all present complexities in study planning. Prominent shared targets include IDH1 R132H and H3 K27M; however, the scale and complexity of trials necessitate the involvement of specialized centers and careful selection of endpoints. Regarding non-muscle-invasive bladder cancer, the development model centers on local immune therapy within the bladder mucosa, employing repeated access and recurrence-based endpoints, as exemplified by BCG and BCG plus cytokine combinations such as Anktiva, which establish the regulatory benchmark [5,6]. Concerning BRCA-associated breast cancer, the initial clinical program should exclude a universal vaccine trial in unaffected carriers. Instead, it should prioritize target discovery and safety evaluation within high-risk cohorts. Should a shared somatic antigen be identified, early clinical evaluations are most likely to involve adjuvant therapy or treatment of minimal residual disease (MRD)-positive cases rather than vaccination of all unaffected carriers (see Table 10).

## 14. Why Do Many Familiar Cancer Antigens Not Support the Central Claim

A definitive boundary must be drawn around the central thesis. HER2 is clinically validated as a therapeutic target and supports immunotherapy concepts, but it is not exclusive to abnormal tissue. MUC1 is frequently altered and overexpressed in many epithelial tumors but also exists as a normal mucin [20]. hTERT is active in many cancers but is not a tumor-specific neoepitope. WT1, survivin, PRAME, NY-ESO-1, mammaglobin, PSMA, and similar antigens may have value in defined therapeutic settings, but they do not meet the same standard as HPV E6 and E7, EBV latent antigens, or mutation-generated peptides.

The cautionary trial record summarized in Section 2.5 reinforces this principle. The MAGRIT phase 3 trial of MAGE-A3 immunotherapy in resected MAGE-A3-positive non-small-cell lung cancer did not enhance disease-free survival [9]. The ACT IV phase 3 trial of the EGFRvIII vaccine, rindopepimut, did not improve survival in patients with newly diagnosed EGFRvIII-positive glioblastoma [10]. PROSTVAC did not demonstrate an improvement in survival in metastatic castration-resistant prostate cancer [11]. Tecemotide failed to meet its primary overall survival endpoint in the START trial [12] for unresectable stage III non-small-cell lung cancer and was not rescued by subsequent analyses [13]. These trials provide evidence that the failure lies not in vaccination per se but in the presence of weak self-antigens, single-antigen designs, bulky or unselected disease, and inadequate biomarker enrichment.

The experience with the MUC1 vaccine in the prevention of colorectal adenoma offers valuable insights from an alternative perspective. In a feasibility study, a 100-mer MUC1 peptide vaccine demonstrated immunogenicity in approximately forty-four percent of vaccinated individuals with a history of advanced adenomas, elicited durable MUC1-specific IgG responses, and established immune memory [86]. The trial indicated that immunoprevention is a viable strategy and that tumor-associated antigens can provoke immune responses in premalignant conditions. MUC1 is not solely a neoantigen expressed exclusively by cancer cells; this example should be cited as evidence that immunoprevention extends beyond the narrow cancer-only epitope paradigm. It should not be interpreted as evidence that a tumor-associated antigen satisfies the strict criterion of being a cancer-specific epitope.

A historical benchmark is the National Cancer Institute antigen prioritization framework [1], which organized candidate cancer antigens based on therapeutic function, immunogenicity, specificity, oncogenicity, prevalence, and other criteria. This framework included numerous tumor-associated antigens, which are beyond the primary focus of this manuscript. It is cited as a historical point of reference illustrating traditional prioritization methods for cancer-vaccine targets, rather than as an endorsement of a strict tumor-only criterion. The current framework adopts a more conservative approach.

Alpha-lactalbumin in breast cancer prevention [21] exemplifies the same principle. Alpha-lactalbumin is normally expressed exclusively during lactation and can be expressed in certain triple-negative breast cancers. This biological behavior supports a controlled tissue-state vaccination concept in specific high-risk populations, such as women who have completed childbearing or prior to a planned prophylactic mastectomy. It, however, does not validate a tumor-specific epitope. The example is pertinent to a breast cancer prevention discussion, with clearly defined safety parameters, and should not be included in any section dedicated solely to strict cancer-targeting approaches.

## 15. Library Governance for a Pre-Manufactured mRNA Vaccine Portfolio

This section introduces a conceptual governance model. The discussion delineates a theoretical framework for the prospective development of a shared tumor-specific mRNA vaccine library; it does not constitute an operational system. Currently, no such library exists in practice. The target passport, retirement rules, and versioned FASTA/GenBank schema, as described herein and in the sequence-anchored reference-design section (Section 17), are intended to serve as a transparent and adaptable framework that any future product development organization may adopt or modify. They do not represent an operational deliverable of this manuscript.

The most credible product concept is not a single universal vaccine but a governed library of shared tumor-specific mRNA modules. The product set is manufactured in advance, quality-controlled, stored, and selected by a diagnostic rule, while the patient receives only the module or modules matching the patient’s tumor antigen, HLA type, viral status, or inherited-syndrome context. This is different from both a fully personalized vaccine and a one-size-fits-all vaccine.

A library member must possess a target passport. This passport must specify the antigen source, the precise molecular event, the applicable tumor type or risk syndrome, the HLA restriction if known, the empirical evidence of natural presentation, the anticipated immune mechanism, the safety evidence pertaining to normal tissue, the preferred clinical setting, the recommended combination therapy, and the escape-monitoring plan. Without this passport, an antigen list remains a discovery inventory rather than a clinically applicable vaccine library.

The library model also addresses the BRCA problem without implying that BRCA itself is an antigen. BRCA carriers may be enrolled in surveillance and discovery programs; if a carrier develops a premalignant or malignant lesion containing a validated library target, a pre-approved mRNA module can be selected. If no validated target is identified, the individual should not receive a generic BRCA vaccine. For KRAS, library governance must account for allele-specific and HLA-specific considerations, since a G12D, G12V, G12R, G12C, or G13D module is not interchangeable with another allele and may be relevant only within specific HLA contexts. For frameshift peptide panels, governance must reflect panel composition, HLA coverage, immunodominance, and recurrence frequency in premalignant and malignant lesions within the target population. For viral oncoprotein modules, eligibility should be limited to tumors or precancers that express the relevant viral antigens, with tumor expression verified prior to module selection. For EBV-associated modules, eligibility should also consider the latency program expressed by the specific EBV-positive disease.

Library members should be subject to retirement rules. A module should be downgraded or removed if later evidence shows poor natural presentation, unacceptable normal-tissue cross-reactivity, low population coverage after HLA filtering, frequent antigen loss under immune pressure, or absence of clinical signal despite strong immunogenicity. A rare module may remain valuable if the disease is aggressive and the target is uniquely clean. A living target atlas is a more realistic objective than a single fixed universal construct.

To make the passport concrete rather than descriptive, Table 11 provides a target-passport template enumerating the required fields for any library module, including accession and version, the precise molecular event, expected translated product, and ORF checksum, normal-tissue and natural-presentation evidence, clinical evidence tier, and a pre-specified retirement trigger.

Retirement is likewise operationalized as an explicit decision structure. Table 12 sets out a module-retirement decision tree whose sequential gates govern whether a module is retained, downgraded, or removed as evidence accrues.

## 16. Limitations and Uncertainties

Several limitations apply across this framework. Firstly, none of the clinical examples cited constitutes an approved indication for a shared-antigen mRNA cancer vaccine; existing published evidence supports mechanisms such as immunogenicity, MRD signals, and intermediate clinical endpoints in small trials but does not establish definitive efficacy. Although some reports indicate differences in survival or recurrence-free survival, these findings are derived from small, often single-arm or non-randomized trials, or from biomarker-defined subgroups within larger studies. Such results necessitate validation in larger randomized trials before their implications for standard care are realized.

Secondly, the distribution of HLA alleles exhibits significant variation across populations with diverse ancestral backgrounds. The majority of published immunopeptidomic and T-cell recognition research has concentrated on HLA contexts, notably HLA-A*02:01, which is over-represented in cohorts of European descent. It is essential that a universally applicable shared mRNA vaccine library incorporates modules and validation data covering the HLA alleles pertinent to the populations it aims to serve. Ensuring equitable coverage is a fundamental development requirement, rather than merely an ancillary consideration.

Third, the strict tumor-only rule cannot always be met in absolute terms; the practical question is whether the relevant peptide–HLA complex is detectable on essential normal tissues at levels that would produce clinically meaningful off-tumor recognition. For some targets, such as KRAS and CALR mutant C-terminal peptides, no evidence has so far been reported of the relevant peptide–HLA complexes in essential normal tissues at current detection limits; this does not prove that off-tumor expression does not occur. Normal-tissue immunopeptidomics remains incomplete; low-level expression and cross-reactive peptide presentation may persist, and the framework therefore requires continuous re-evaluation as detection methods improve.

Fourth, target identification is necessary but not sufficient. Tumor immune-evasion mechanisms such as HLA loss, beta-2 microglobulin deficiency, antigen presentation defects, T-cell exclusion, and suppressive myeloid biology can undermine even an optimally targeted vaccine. Many of the development programs referenced will require combinations including checkpoint blockade, tumor-microenvironment modification, chemotherapy, radiotherapy, or targeted therapy. The effects of such combinations must be empirically tested, not presumed.

Fifth, the sequence-design section provides representative reference designs derived computationally from cited canonical UniProt or NCBI RefSeq protein entries. Independent residue-by-residue verification against the cited canonical accessions is required prior to laboratory implementation of any construct, and all clinical or preclinical work must include independent sequence retrieval and verification. The H3 K27M reference tile was corrected during revision so that its central motif matches the clinical H3K27M p14–40 peptide, and the associated ORF and table entries were updated. Sixth, sponsor, registry, and trade-press sources were used only for regulatory or trial-status facts and not as evidence of clinical efficacy; regulatory milestones such as IND clearance, Fast Track designation, first patient dosed, or recruiting or ongoing status indicate permission to investigate or trial progress and do not imply therapeutic benefit.

## 17. Sequence-Anchored Reference Designs and Computational Verification

This section enumerates representative human-codon-compatible open reading frames (ORFs) for the fifteen sequence-anchored candidates, systematically arranged across thirteen target candidates and four superclasses. Candidate 2 (Merkel cell polyomavirus) is presented solely at the design-specification level due to the inability to authenticate a canonical accession-anchored ORF within the scope of this manuscript. The CALR exon 9 frameshift is represented by a single construct because the type 1 and type 2 mutations converge on one shared novel C-terminus. Consequently, the count of sequence-anchored constructs is fifteen, with an additional placeholder count of one, resulting in a total of sixteen construct modules.

Each entry below identifies the source UniProt accession (or NCBI RefSeq where specified), the encoded antigenic region in single-letter amino acid code, and a representative human-codon-compatible DNA sequence for the open reading frame (ORF). Each ORF commences with an ATG start codon at the 5′ end and concludes with a TGA, TAA, or TAG stop codon at the 3′ end. The notation of the encoded protein starts with the methionine encoded by ATG; in instances where the source protein region does not naturally begin with methionine, the methionine is added (or, as indicated, replaces the natural first residue) as an artificial start codon. These coding sequences use common human codons to illustrate a translatable design; they are not the product of a defined codon-optimization pipeline. No specific optimization algorithm, codon-usage table, GC-content target, motif-avoidance rule, or repeat-avoidance constraint was applied, and any final clinical construct would require formal codon optimization under a documented method and species table. The sequences are therefore described as representative human-codon-compatible coding sequences rather than optimized clinical constructs.

All fifteen sequence-anchored ORFs presented in this section were checked computationally in two stages during preparation. Stage 1: each claimed encoded protein was compared against the canonical UniProt or NCBI RefSeq record (or, for the mutant frameshift and fusion-junction candidates, against the literature-defined mutant sequence) with the documented mutation, attenuation, or junction applied in silico. For the CALR construct, the encoded novel C-terminus was compared against the consensus 36-residue mutant sequence shared by the type 1 and type 2 mutations, as reported by Klampfl et al. and used as the CALRLong36 vaccine immunogen. Stage 2: each ORF was translated under the standard human genetic code, and the resulting protein was compared residue-by-residue to the claimed encoded protein. This internal check is described for transparency and does not replace independent verification; any laboratory or clinical use requires independent residue-by-residue re-derivation of every construct from the cited canonical accession. The H3 K27M reference tile was corrected during revision to align its central motif with the clinical H3K27M p14–40 27-mer (SAPST rather than the previously stated SAPAT); the H3 K27M ORF, translated protein, construct length, and the corresponding entries in Table 13 and Table 14 were updated accordingly.

The ORFs are reference designs intended to illustrate the encoded antigen content and the location of the relevant driver mutation, frameshift, insertion-altered C-terminus, fusion junction, or viral-oncoprotein safety substitution. They are not validated clinical-grade sequences. Independent residue-by-residue verification against the cited canonical accession is required before laboratory implementation. Verification URLs are listed in Table 15.

Candidate 1. HPV E6/E7 (HPV16 and HPV18)

Four sequence-anchored ORFs, two from HPV16 and two from HPV18, were each engineered as an attenuated antigenic reference design intended to retain antigen content while reducing the residual transforming activity of the encoded protein. The two HPV16 attenuations follow established literature: a C-terminal truncation encompassing the PDZ-binding motif of E6 (HPV16 E6 residues 152–158, TRRETQL, which contains the terminal ETQL PDZ-binding motif) [87,88], and a substitution at residue C24 (LXCXE motif) to G24 that disrupts the retinoblastoma-binding pocket of E7 [89]. The HPV18 attenuations parallel the HPV16 design: the HPV18 E6 truncation encompasses the PDZ-binding motif (HPV18 E6 residues 153–158, RRETQV, which contains the terminal ETQV PDZ-binding motif), and the HPV18 E7 LXCXE cysteine is substituted to glycine. These are attenuation concepts, not experimentally validated non-transforming vaccine inserts. PDZ-binding-motif deletion removes only one of several oncogenic activities of high-risk E6, which also inactivates p53, blocks apoptosis, activates telomerase, and disrupts cell adhesion and polarity; the constructs should therefore be described as PDZ-binding-motif-deleted E6 antigenic modules rather than as fully non-transforming unless additional p53/E6AP-disrupting substitutions and experimental transformation assays validate loss of oncogenic activity. Likewise, the E7 LXCXE single-residue substitution should be regarded as an LXCXE-disrupted reference design for which attenuation is expected but not experimentally validated as fully non-transforming; the E7 LXCXE motif is the principal target for retinoblastoma-binding disruption, and a literature-validated detoxified E7 design typically introduces multiple substitutions in the pRb-binding region, so any specific clinical construct may require additional substitutions for full attenuation.

Candidate 1a. HPV16 E6 (attenuated reference design; PDZ-binding-motif deletion at C-terminus)

Source: UniProt P03126 (HPV16 E6).

Encoded antigenic protein (151 aa; canonical HPV16 E6 residues 1–151, with the natural C-terminal residues 152–158 (TRRETQL, the PDZ-binding motif) deleted): MHQKRTAMFQDPQERPRKLPQLCTELQTTIHDIILECVYCKQQLLRREVYDFAFRDLCIVYRDGNPYAVCDKCLKFYSKISEYRHYCYSLYGTTLEQQYNKPLCDLLIRCINCQKPLCPEEKQRHLDKKQRFHNIRGRWTGRCMSCCRSSR

Representative ORF (456 bp including ATG and TGA): ATGCACCAGAAGAGAACCGCCATGTTCCAGGACCCCCAGGAGAGACCCAGAAAGCTGCCC CAGCTGTGCACCGAGCTGCAGACCACCATCCACGACATCATCCTGGAGTGCGTGTACTGC AAGCAGCAGCTGCTGAGAAGAGAGGTGTACGACTTCGCCTTCAGAGACCTGTGCATCGTG TACAGAGACGGCAACCCCTACGCCGTGTGCGACAAGTGCCTGAAGTTCTACAGCAAGATC AGCGAGTACAGACACTACTGCTACAGCCTGTACGGCACCACCCTGGAGCAGCAGTACAAC AAGCCCCTGTGCGACCTGCTGATCAGATGCATCAACTGCCAGAAGCCCCTGTGCCCCGAG GAGAAGCAGAGACACCTGGACAAGAAGCAGAGATTCCACAACATCAGAGGCAGATGGACC GGCAGATGCATGAGCTGCTGCAGAAGCAGCAGATGA

Candidate 1b. HPV16 E7 (attenuated reference design; LXCXE C-to-G substitution at position 24)

Source: UniProt P03129 (HPV16 E7).

Encoded antigenic protein (98 aa; full-length E7 with C24G substitution to disrupt the retinoblastoma-binding pocket; all other residues unchanged from canonical): MHGDTPTLHEYMLDLQPETTDLYGYEQLNDSSEEEDEIDGPAGQAEPDRAHYNIVTFCCKCDSTLRLCVQSTHVDIRTLEDLLMGTLGIVCPICSQKP

Representative ORF (297 bp including ATG and TGA): ATGCACGGCGACACCCCCACCCTGCACGAGTACATGCTGGACCTGCAGCCCGAGACCACC GACCTGTACGGCTACGAGCAGCTGAACGACAGCAGCGAGGAGGAGGACGAGATCGACGGC CCCGCCGGCCAGGCCGAGCCCGACAGAGCCCACTACAACATCGTGACCTTCTGCTGCAAG TGCGACAGCACCCTGAGACTGTGCGTGCAGAGCACCCACGTGGACATCAGAACCCTGGAG GACCTGCTGATGGGCACCCTGGGCATCGTGTGCCCCATCTGCAGCCAGAAGCCCTGA

Candidate 1c. HPV18 E6 (attenuated reference design; C-terminal PDZ-binding-motif deletion)

Source: UniProt P06463 (HPV18 E6).

Encoded antigenic protein (152 aa; canonical HPV18 E6 residues 1–152, with the natural C-terminal residues 153–158 (RRETQV, the PDZ-binding motif) deleted): MARFEDPTRRPYKLPDLCTELNTSLQDIEITCVYCKTVLELTEVFEFAFKDLFVVYRDSIPHAACHKCIDFYSRIRELRHYSDSVYGDTLEKLTNTGLYNLLIRCLRCQKPLNPAEKLRHLNEKRRFHNIAGHYRGQCHSCCNRARQERLQR

Representative ORF (459 bp including ATG and TGA): ATGGCCAGATTCGAGGACCCCACCAGAAGACCCTACAAGCTGCCCGACCTGTGCACCGAG CTGAACACCAGCCTGCAGGACATCGAGATCACCTGCGTGTACTGCAAGACCGTGCTGGAG CTGACCGAGGTGTTCGAGTTCGCCTTCAAGGACCTGTTCGTGGTGTACAGAGACAGCATC CCCCACGCCGCCTGCCACAAGTGCATCGACTTCTACAGCAGAATCAGAGAGCTGAGACAC TACAGCGACAGCGTGTACGGCGACACCCTGGAGAAGCTGACCAACACCGGCCTGTACAAC CTGCTGATCAGATGCCTGAGATGCCAGAAGCCCCTGAACCCCGCCGAGAAGCTGAGACAC CTGAACGAGAAGAGAAGATTCCACAACATCGCCGGCCACTACAGAGGCCAGTGCCACAGC TGCTGCAACAGAGCCAGACAGGAGAGACTGCAGAGATGA

Candidate 1d. HPV18 E7 (attenuated reference design; LXCXE C-to-G substitution at position 27)

Source: UniProt P06788 (HPV18 E7).

Encoded antigenic protein (105 aa; full-length HPV18 E7 with C27G substitution to disrupt the LXCXE retinoblastoma-binding pocket; all other residues unchanged from canonical): MHGPKATLQDIVLHLEPQNEIPVDLLGHEQLSDSEEENDEIDGVNHQHLPARRAEPQRHTMLCMCCKCEARIKLVVESSADDLRAFQQLFLNTLSFVCPWCASQQ

Representative ORF (318 bp including ATG and TGA): ATGCACGGCCCCAAGGCCACCCTGCAGGACATCGTGCTGCACCTGGAGCCCCAGAACGAG ATCCCCGTGGACCTGCTGGGCCACGAGCAGCTGAGCGACAGCGAGGAGGAGAACGACGAG ATCGACGGCGTGAACCACCAGCACCTGCCCGCCAGAAGAGCCGAGCCCCAGAGACACACC ATGCTGTGCATGTGCTGCAAGTGCGAGGCCAGAATCAAGCTGGTGGTGGAGAGCAGCGCC GACGACCTGAGAGCCTTCCAGCAGCTGTTCCTGAACACCCTGAGCTTCGTGTGCCCCTGG TGCGCCAGCCAGCAGTGA

Candidate 2. Merkel cell polyomavirus T antigens (design specification only)

Sequence-anchored ORFs for the MCPyV large and small T antigens were not finalized within the scope of this manuscript because no single canonical accession could be authenticated for a non-transforming antigenic construct without further laboratory verification. The conceptual design would retain the antigenic regions of the truncated tumor-derived large T antigen and the small T antigen [16,96], with substitutions to remove the LXCXE motif of the large T antigen (preventing retinoblastoma binding) and substitutions in the small T antigen to disrupt PP2A binding. UniProt entries B6DVY9 (large T antigen) and B0G0V7 (small T antigen) are cited as illustrative source records only; no non-transforming, sequence-anchored MCPyV ORF is provided or authenticated in this manuscript. A definitive ORF for clinical use should be constructed from a non-transforming antigenic subdomain after empirical validation of antigen content and absence of transforming activity, with the canonical accession re-confirmed against the live UniProtKB record at the time of construction. This candidate is therefore a conceptual placeholder and is excluded from the count of sequence-anchored constructs.

Candidate 3. KRAS G12D

Source: UniProt P01116 (KRAS, isoform 4B).

Encoded antigenic protein (37 aa; residues 1–37 of KRAS with G12D substitution; the wild-type residue G at position 12 is replaced with D): MTEYKLVVVGADGVGKSALTIQLIQNHFVDEYDPTIE

Representative ORF (114 bp including ATG and TGA): ATGACCGAGTACAAGCTGGTGGTGGTGGGCGCCGACGGCGTGGGCAAGAGCGCCCTGACC ATCCAGCTGATCCAGAACCACTTCGTGGACGAGTACGACCCCACCATCGAGTGA

Candidate 4. KRAS G12V

Source: UniProt P01116 (KRAS, isoform 4B).

Encoded antigenic protein (37 aa; residues 1–37 of KRAS with G12V substitution): MTEYKLVVVGAVGVGKSALTIQLIQNHFVDEYDPTIE

Representative ORF (114 bp including ATG and TGA): ATGACCGAGTACAAGCTGGTGGTGGTGGGCGCCGTGGGCGTGGGCAAGAGCGCCCTGACC ATCCAGCTGATCCAGAACCACTTCGTGGACGAGTACGACCCCACCATCGAGTGA

Candidate 5. KRAS G12R

Source: UniProt P01116 (KRAS, isoform 4B).

Encoded antigenic protein (37 aa; residues 1–37 of KRAS with G12R substitution): MTEYKLVVVGARGVGKSALTIQLIQNHFVDEYDPTIE

Representative ORF (114 bp including ATG and TGA): ATGACCGAGTACAAGCTGGTGGTGGTGGGCGCCAGAGGCGTGGGCAAGAGCGCCCTGACC ATCCAGCTGATCCAGAACCACTTCGTGGACGAGTACGACCCCACCATCGAGTGA

Candidate 6. KRAS G12C

Source: UniProt P01116 (KRAS, isoform 4B).

Encoded antigenic protein (37 aa; residues 1–37 of KRAS with G12C substitution): MTEYKLVVVGACGVGKSALTIQLIQNHFVDEYDPTIE

Representative ORF (114 bp including ATG and TGA): ATGACCGAGTACAAGCTGGTGGTGGTGGGCGCCTGCGGCGTGGGCAAGAGCGCCCTGACC ATCCAGCTGATCCAGAACCACTTCGTGGACGAGTACGACCCCACCATCGAGTGA

Candidate 7. KRAS G13D

Source: UniProt P01116 (KRAS, isoform 4B).

Encoded antigenic protein (37 aa; residues 1–37 of KRAS with G13D substitution): MTEYKLVVVGAGDVGKSALTIQLIQNHFVDEYDPTIE

Representative ORF (114 bp including ATG and TGA): ATGACCGAGTACAAGCTGGTGGTGGTGGGCGCCGGCGACGTGGGCAAGAGCGCCCTGACC ATCCAGCTGATCCAGAACCACTTCGTGGACGAGTACGACCCCACCATCGAGTGA

Direct biological support for KRAS G12D immunogenicity in vivo comes from adoptive transfer of HLA-C*08:02-restricted T cells specific for KRAS G12D, which produced regression of metastatic pancreatic ductal adenocarcinoma in a single patient [17]. Mutant KRAS is HLA-restricted, and a pre-manufactured product is a mutation-by-HLA library rather than a single construct.

Candidate 8. IDH1 R132H

Source: UniProt O75874 (IDH1).

Encoded antigenic protein (21 aa; artificial start methionine + canonical IDH1 residues 123–142 with R132H substitution; this 20-mer tile corresponds to the IDH1-vac peptide used in published phase 1 trials [59,60], and the mutation is centrally placed within a class II-restricted tile): MGWVKPIIIGHHAYGDQYRAT.

Representative ORF (66 bp including ATG and TGA): ATGGGCTGGGTGAAGCCCATCATCATCGGCCACCACGCCTACGGCGACCAGTACAGAGCC ACCTGA.

The peptide tile encompasses the residue R132H mutation at position 11 of the 21-aa tile (the canonical position 132 of IDH1, which is R in wild-type and H in the mutant). Class II-restricted CD4+ T-cell responses against IDH1 R132H have been demonstrated preclinically [59] and clinically [60].

Candidate 9. Histone H3 K27M

Source: UniProt P84243, histone H3.3, encoded by H3-3A/H3F3A and H3-3B/H3F3B.

Encoded antigenic protein (21 aa; artificial start methionine replacing the natural L21 of canonical H3.3, followed by residues 22–41, with the K28M substitution applied at position 8 of the tile; the mutation labeled “K27M” by clinical convention corresponds to residue 28 in the canonical UniProt sequence in which the initial methionine is counted): MATKAARMSAPSTGGVKKPHR.

Representative ORF (66 bp including ATG and TGA): ATGGCCACCAAGGCCGCCAGAATGAGCGCCCCCAGCACCGGCGGCGTGAAGAAGCCCCAC AGATGA.

The peptide tile encompasses the K27M (canonical residue 28) mutation at position 8 of the 21-aa tile. The tile corresponds to the C-terminal portion of the clinical H3K27M p14–40 27-mer (KAPRKQLATKAARMSAPSTGGVKKPHR) used in the H3K27M-vac long-peptide vaccine, with the mutant methionine and the downstream SAPST motif preserved; the natural N-terminal residues p14–20 of the clinical 27-mer are not included in this shorter reference tile, and a specific clinical construct may instead encode the full 27-mer. Class II-restricted T-cell responses against H3 K27M have been demonstrated in early-phase trials [61]. Reports of HLA-A*02:01-restricted class I targeting in vivo have not been confirmed [91], and the primary clinical focus is class II-restricted CD4+ T-cell biology [90].

Candidate 10. NPM1 mutant Type A (insertion-altered C-terminus)

Source: UniProt P06748 (NPM1).

Encoded antigenic protein (48 aa; a methionine-initiated tile beginning at the natural internal methionine at residue 251 of NPM1 and extending to the C-terminal lysine of the mutant protein at residue 298). The tile comprises the wild-type NPM1 sequence from residue 251 through residue 287, followed by the 11-residue novel C-terminus (CLAVEEVSLRK) generated by the +4 nucleotide insertion within exon 12, which replaces the wild-type C-terminal heptapeptide WQWRKSL and shifts the reading frame. The novel C-terminal lysine immediately precedes the natural stop codon of the mutant transcript:

MQASIEKGGSLPKVEAKFINYVKNCFRMTDQEAIQDLCLAVEEVSLRK.

Representative ORF (147 bp including ATG and TGA): ATGCAGGCCAGCATCGAGAAGGGCGGCAGCCTGCCCAAGGTGGAGGCCAAGTTCATCAAC TACGTGAAGAACTGCTTCAGAATGACCGACCAGGAGGCCATCCAGGACCTGTGCCTGGCC GTGGAGGAGGTGAGCCTGAGAAAGTGA.

The natural internal methionine of NPM1 at residue 251 is used as the start codon for the encoded antigenic region. The tile comprises wild-type residues 251 through 287 (37 residues) followed by the 11-residue novel C-terminus (residues 288 through 298 of the mutant protein), so the encoded protein is 48 residues in total. The 147 bp ORF length corresponds to 144 bp of coding sequence (3 bp × 48 amino acids, counting the methionine encoded by the natural internal ATG at residue 251 as the first of the 48 residues) plus the 3 bp TGA stop codon. The wild-type C-terminal heptapeptide WQWRKSL is replaced by the novel 11-residue sequence CLAVEEVSLRK, which is the recurrent neoepitope of Type A NPM1-mutant AML. Mutant NPM1 C-terminal peptides have been directly identified on AML cells by immunopeptidomics [19], and TCR-engineered T cells specific for mutant NPM1 have demonstrated mutant-specific cytotoxicity against AML cells in preclinical studies [92]. The construct is presented as a representative reference and is not a validated clinical sequence; clinical implementation requires independent verification against the canonical NPM1 mutant Type A protein record.

Candidate 11. CALR exon 9 frameshift

Source: UniProt P27797 (CALR) plus the recurrent exon 9 frameshift-derived novel C-terminus described in Klampfl et al. 2013 [93].

CALR mutations in essential thrombocythemia and primary myelofibrosis are insertions or deletions within exon 9 that all induce a +1 frameshift into the same alternative reading frame, generating a shared novel positively charged C-terminus and loss of the wild-type KDEL endoplasmic-reticulum-retention motif. The two most common variants, type 1 (52 bp deletion, p.L367fs*46) and type 2 (5 bp TTGTC insertion, p.K385fs*47), together with more than fifty rarer type 1-like and type 2-like indels, converge on an identical novel 36-amino-acid C-terminus. What differs between the variants is the extent to which the upstream wild-type acidic residues are retained (type 1 eliminates nearly all of them; type 2 retains approximately half), not the length or composition of the novel C-terminal neoantigen itself. Because the shared neoantigen is the common 36-residue C-terminus, a single sequence-anchored construct represents the CALR target class for both type 1 and type 2 disease [18,93].

Encoded antigenic protein (37 aa; artificial start methionine plus the shared 36-aa novel C-terminus common to the type 1 (c.1099_1150del52) and type 2 (c.1154_1155insTTGTC) mutations and to other CALR exon 9 indels, corresponding to the CALRLong36 immunogen described by Klampfl et al. 2013 [93]): MRMRRMRRTRRKMRRKMSPARPRTSCREACLQGWTEA

Representative ORF (114 bp including ATG and TGA): ATGAGAATGAGAAGAATGAGAAGAACCAGAAGAAAGATGAGAAGAAAGATGAGCCCCGCC AGACCCAGAACCAGCTGCAGAGAGGCCTGCCTGCAGGGCTGGACCGAGGCCTGA

The 36-residue novel C-terminus is identical across type 1, type 2, and the rarer type 1-like and type 2-like CALR indels, and has been identified as a shared neoantigen recognized by CD4+ and CD8+ T cells [18]. A single construct therefore represents the CALR-mutant population. Preclinical work on a CALR mutant peptide vaccine indicates that the MHC class I down-regulation seen in CALR-mutant tumors can be partially counteracted by optimized vaccine design [63]; a synthetic long-peptide form of this same C-terminus (CALRLong36) has been administered to patients with CALR-mutant myeloproliferative neoplasms.

Candidate 12. EWS-FLI1 fusion (Type 1 junction)

Source: NCBI RefSeq EWSR1 (NP_001157610.1) and FLI1 (NP_002008.1); the fusion junction is generated by the t(11;22)(q24;q12) chromosomal translocation [94]. The Type 1 junction is the most common, joining EWSR1 exon 7 to FLI1 exon 6 [95].

Encoded antigenic protein (22 aa; methionine-initiated tile spanning the EWS-FLI1 Type 1 junction): MSSYGQQNPSYASTGDSVNSLY.

Representative ORF (69 bp including ATG and TGA): ATGAGCAGCTACGGCCAGCAGAACCCCAGCTACGCCAGCACCGGCGACAGCGTGAACAGC CTGTACTGA.

The EWS-FLI1 Type 1 junction is the most common breakpoint in Ewing sarcoma. The construct is presented as a sequence-anchored fusion-junction reference design; the cited references authenticate the fusion structure and the Type 1 transcript and its prognostic context but do not by themselves establish that the selected junction tile is naturally processed and presented as a peptide–HLA target or that it elicits tumor-cell recognition by T cells. Natural peptide–HLA presentation and immunogenicity therefore remain to be demonstrated for the selected junction tile, and under the strict rule adopted here, EWS-FLI1 should be treated as sequence-defined but not presentation-validated. Alternative breakpoints (Type 2 and others) generate different junctional peptide sequences. Breakpoint heterogeneity limits the universality of any single junction-spanning construct, and a complete library member should account for the principal breakpoints in the target population.

Candidate 13. BCR-ABL fusion (b3a2 junction)

Source: NCBI RefSeq BCR (NP_004318.3) and ABL1 (NP_005148.2); the b3a2 fusion junction is generated by the t(9;22)(q34;q11) Philadelphia translocation. The b3a2 junction joins BCR exon e14 to ABL1 exon 2 and corresponds to the p210 BCR-ABL protein in chronic myeloid leukemia.

Encoded antigenic protein (18 aa; methionine-initiated tile spanning the BCR-ABL b3a2 junction): MATGFKQSSKALQRPVAS.

Representative ORF (57 bp including ATG and TGA): ATGGCCACCGGCTTCAAGCAGAGCAGCAAGGCCCTGCAGAGACCCGTGGCCAGCTGA.

Direct immunopeptidomic evidence indicates that the BCR-ABL b3a2 junction can be presented on HLA molecules of leukemic cells, providing the basis for T-cell recognition [84]. The dominance of tyrosine kinase inhibitor therapy in CML has limited clinical development of BCR-ABL fusion-junction vaccines, but the molecule remains a sequence-anchored reference case for fusion-junction vaccinology.

## 18. Source-Anchoring Summary Table

Computational sequence verification (Table 14) confirms only that each encoded protein and ORF is internally correct relative to the canonical record and the documented event; it does not establish that the encoded peptide is naturally processed and presented as a peptide–HLA complex on tumor cells. To keep these two distinct properties from being read as equivalent, Table 14a separates them explicitly, stating for each construct the canonical accession, the mutant or junction event, whether the translated ORF was residue-level confirmed against the claimed protein, and, as a separate column, whether natural peptide–HLA presentation has been experimentally demonstrated. A construct can be sequence-correct yet presentation-unvalidated, and several entries are exactly that.

### Sequence Verification URLs

The following URLs direct readers to the canonical UniProt or NCBI RefSeq records used as source accessions for each sequence-anchored candidate. Each ORF is a representative reference design; before laboratory implementation of any specific candidate, the encoded antigen sequence should be verified residue-by-residue against the canonical record at the URL provided.

## 19. Conclusions

A scientifically credible, non-personalized mRNA cancer vaccine is not considered a universal solution for cancer. Instead, it is a component of a carefully curated library of shared tumor-specific mRNA constructs targeting well-defined molecular subgroups. These targets are characterized by their absence from essential normal tissues, their recurrent presence within the specified subgroup, their natural presentation on tumor HLA molecules, and their sufficient significance to the tumor to prevent immune escape. The leading candidates currently include HPV E6 and E7 for HPV-associated cancers; mutant KRAS (G12D, G12V, G12R, G12C, and G13D) for pancreatic and other KRAS-mutant cancers, especially in contexts of adjuvant therapy and minimal residual disease; panels of Lynch syndrome and MSI-derived frameshift peptides for cancer prevention in mismatch-repair-deficient biological systems; IDH1 R132H and H3 K27M for particular glioma subgroups; NPM1 insertion-altered C-terminal neoantigens and CALR exon 9 frameshift neoantigens for certain hematologic malignancies; MCPyV T antigens for virus-positive Merkel cell carcinoma; and EBV latent antigens for specific EBV-associated malignancies, including nasopharyngeal carcinoma.

The historical record supports the selection logic for some classes and constrains the interpretation of others; it does not establish proof of benefit for any shared, pre-manufactured mRNA cancer vaccine. Preventive HBV and HPV vaccinations have demonstrated that immunization can avert cancer at the population level. BCG has evidenced that local immune stimulation can revolutionize the management of recurrence in non-muscle-invasive bladder cancer. T-VEC has proven that oncolytic virus therapy can attain approval as a treatment for cancer in anatomically favorable melanoma lesions. The failures of vaccines targeting MAGE-A3, EGFRvIII, PSA, and MUC1 in late-stage disease are equally informative and provide valuable guidance.

BRCA-associated breast cancer should be regarded as a corrective case, not as an existing shared-antigen target. BRCA carriers constitute a significant risk-enriched population for the discovery of therapeutic targets; currently, no universally recognized, cancer-specific BRCA-associated antigen has been validated. Any future vaccine targeting BRCA-associated cancer must focus on validated downstream somatic neoantigens, junctions, or splice events, or alternatively, on precisely defined tissue-restricted preventive antigens with transparent safety profiles.

In all contexts, an immune response is considered evidence of a mechanism rather than evidence of cancer prevention or survival benefit. Prevention, interception, adjuvant therapy, minimal residual disease (MRD) therapy, and active-disease treatment each necessitate distinct endpoint logic, and evidence from one setting should not be overgeneralized to another. The manuscript delineates two tiers of evidence: a proof-of-mechanism tier, comprising limited immunopeptidomic detection in selected cell lines or models, single-arm phase 1 or phase 2 immune-response data, and notable single-patient case reports such as the KRAS G12D adoptive T-cell case, which support the premise that a target class can, in principle, induce antigen-specific immunity; and a proof-of-benefit tier, comprising randomized controlled data with clinical endpoints, replicated and ideally with regulatory approval, that would justify clinical adoption. Currently, none of the shared tumor-specific vaccine classes discussed in this manuscript possesses proof-of-benefit-tier evidence as a commonly available pre-manufactured mRNA product. Classes supported solely by limited immunopeptidomics data for select alleles, a small single-arm trial, or significant case reports should be classified within the proof-of-mechanism tier, and their inclusion within this framework is supported accordingly. The mRNA platform is well-suited for library development due to its modular update feasibility; tumor immunology, rather than platform chemistry, will determine clinical success. The most credible near-term approach involves a modular library of non-personalized vaccines tailored to molecular subgroups, HLA context, and clinical setting, developed under explicit validation criteria and combined as necessary with checkpoint blockade or other immune modulation.

## Figures and Tables

**Table 1:** Target classes evaluated under the strict cancer-cell-only rule.

Target Class	Representative Examples	Cancer-Only Status	Recommended Wording
Viral oncoprotein	HPV16 and HPV18 E6 and E7; MCPyV large and small T antigens; EBV latent antigens	High in defined virus-positive tumors; greater clinical maturity for HPV than for MCPyV or EBV	Best pre-manufactured therapeutic class when tumor expression and natural presentation are verified; therapeutic vaccines do not replace prophylactic infection vaccines
Recurrent driver neoepitope	KRAS G12D, G12V, G12R, G12C, G13D; IDH1 R132H; H3 K27M	High for mutation-positive cells but HLA-restricted	Strong for molecularly defined subgroups; requires allele- and HLA-aware product libraries
Mismatch-repair-derived recurrent abnormal peptides	Lynch- and MSI-associated frameshift peptide panels (TGFBR2, BAX, ACVR2A, MSH3 and related coding microsatellites)	High when absent from the normal proteome and naturally presented	Strongest current experimental prevention and interception model; clinical cancer-incidence reduction remains unproven
Hematologic mutation-derived neoantigens	Mutant NPM1 C-terminal peptides; mutant CALR exon 9 C-terminus	High when tumor-specific and naturally presented	Distinguish NPM1 insertion-altered C-terminus from MSI-style frameshift biology; strongest MRD-focused settings
Fusion-junction peptides	BCR-ABL; EWS-FLI1; selected kinase and transcription-factor fusions	High at the junction, but breakpoint and HLA variability matter	Useful in selected cancers; not broadly applicable across unrelated tumor types
Tumor-associated antigen	HER2, MUC1, hTERT, WT1, PSMA, survivin, NY-ESO-1, mammaglobin, alpha-lactalbumin	Usually not strictly cancer-only	May be valuable therapeutic or prevention research targets but do not authenticate the shared tumor-specific vaccine claim; multiple late-stage failures inform design

**Table 2:** Vaccine target candidates, variants, constructs, and associated cancers. Each entry is a design-stage vaccine target candidate, not a validated or approved vaccine product.

#	Candidate	Variants	Constructs	Associated Cancers
1	HPV E6/E7	HPV16 E6, HPV16 E7, HPV18 E6, HPV18 E7	4 sequence-anchored	Cervical, anal, oropharyngeal/HNSCC, vulvar, vaginal, and penile cancers
2	MCPyV (conceptual placeholder)	LT, ST	1 placeholder candidate (LT and ST at design-specification level only)	Virus-positive Merkel cell carcinoma
3	KRAS G12D	one minigene	1 sequence-anchored	Pancreatic ductal adenocarcinoma; colorectal; lung adenocarcinoma
4	KRAS G12V	one minigene	1 sequence-anchored	Pancreatic ductal adenocarcinoma; colorectal; lung adenocarcinoma
5	KRAS G12R	one minigene	1 sequence-anchored	Pancreatic ductal adenocarcinoma (predominant)
6	KRAS G12C	one minigene	1 sequence-anchored	Non-small-cell lung cancer; selected colorectal
7	KRAS G13D	one minigene	1 sequence-anchored	Colorectal cancer (predominant); selected lung
8	IDH1 R132H	one tile	1 sequence-anchored	Diffuse IDH1-mutant glioma (WHO grade 2–4 astrocytoma)
9	H3 K27M	one tile	1 sequence-anchored	Diffuse midline glioma, including diffuse intrinsic pontine glioma (DIPG)
10	NPM1 Type A	one tile	1 sequence-anchored	NPM1-mutated acute myeloid leukemia
11	CALR	Type 1 and Type 2 (shared C-terminus)	1 sequence-anchored	Essential thrombocythemia; primary myelofibrosis (CALR-mutant MPN)
12	EWS-FLI1	Type 1 junction	1 sequence-anchored	Ewing sarcoma
13	BCR-ABL	b3a2 junction	1 sequence-anchored	Chronic myeloid leukemia; Philadelphia-positive acute lymphoblastic leukemia
Total			15 sequence-anchored + 1 conceptual placeholder = 16 construct modules	

**Table 3:** BRCA-related claims and corrected wording. The evidence-status and recommended-wording entries are derived from the sources cited in the corresponding text of Section 5, principally the BRCA cancer-risk and genetic-testing references [27,28], the exploratory computational BRCA1 shared-neoantigen analysis [29], the prophylactic breast-vaccination and alpha-lactalbumin studies [21,30], and the INO-5401 carrier trial registry entry [31]; the wording reflects the authors’ synthesis of these sources under the strict cancer-cell-only rule rather than any single primary claim.

Claim	Evidence Status	Recommended Wording
The inherited BRCA variant itself is a tumor-only epitope.	Incorrect	Inherited BRCA variants are germline risk markers present in normal cells and should not be treated as vaccine antigens.
All BRCA carriers share a future cancer antigen.	Not validated	No universal BRCA-wide tumor-only epitope has been validated; exploratory computational subgroup-specific candidates do not establish a clinically usable vaccine target.
BRCA carriers are useful for prevention-vaccine research.	Correct	BRCA carriers are a high-risk cohort for target discovery, interception studies, and carefully bounded tissue-restricted vaccine trials.
Alpha-lactalbumin, hTERT, PSMA, or WT1 are breast prevention candidates under the strict cancer-only rule.	Outside the strict cancer-only framework	These are tissue-restricted or tumor-associated antigens, not strict tumor-specific neoepitopes. They may have research value as tissue-restricted prevention concepts in narrowly defined high-risk cohorts with explicit safety tradeoffs, and should be presented under that label and not as shared tumor-specific vaccines under the central rule of this manuscript.
A BRCA-associated shared vaccine library could be developed in the future.	Speculative research direction	There is currently no validated shared somatic neoantigen class in BRCA-associated disease. Any future library would require discovery of recurrent downstream somatic targets, demonstrated natural presentation on tumor HLA, normal-tissue safety, and clinical validation. The probability that a broad BRCA library reaches practice is unknown and may be low.

**Table 4:** Prevention and interception hierarchy.

Approach	Evidence Category	Why It Matters	Most Suitable Setting
HPV or HBV infection prevention	Established public-health prevention	Prevents viral infection or chronic disease that can lead to cancer	Population vaccination before exposure
Local immune therapy (BCG and BCG combinations)	Long-established standard with active combination development	Direct local immune activation in accessible mucosa	Non-muscle-invasive bladder cancer
Lynch/MSI shared frameshift-peptide vaccination	Strongest current experimental tumor-interception model	Risk mechanism creates recurrent abnormal peptide classes in premalignant or malignant tissue	Lynch carriers; endpoints should include safety, immune response, adenomas or advanced neoplasia, and eventually cancer incidence
BRCA-associated breast cancer	Risk-cohort discovery model; not a proven tumor-only target	Inherited risk is not equivalent to a shared tumor-specific antigen	High-risk carriers for discovery sampling, tissue biomarkers, and carefully bounded prevention concepts
Tumor-associated antigen prevention	Biologically plausible but not strictly cancer-only	Targets may be overexpressed in cancer but are still present in normal tissues	High-risk cohorts only with a strong safety rationale
Adjuvant or MRD vaccination	Fastest practical efficacy test for many targets	Residual disease is present or likely, and recurrence can be measured	PDAC, CRC, AML, melanoma, HPV-positive HNSCC, and other molecularly defined settings

**Table 5:** Priority Atlas for Shared Tumor-Specific Vaccine Development. The priority interpretations in the rightmost column are informed by the authors’ judgment, based on currently available biological and early clinical evidence. These interpretations are not evidence-based rankings and should not be construed as predictions of clinical success or regulatory approval.

Target Family	Cancer Setting	Why It Fits or Fails the Strict Rule	Most Useful Early Endpoint	Priority Interpretation
HPV16/18 E6 and E7	Cervical, anal, oropharyngeal/HNSCC, and other HPV-driven cancers	Foreign viral oncoproteins with high tumor specificity in HPV-positive tumors	Measurable disease, adjuvant recurrence prevention, or combinations with checkpoint blockade	Conceptual priority for pre-manufactured therapeutic vaccination based on target biology; clinical efficacy unproven. Platform examples in development: shared mRNA (BNT113, investigational), peptide (ISA101), DNA (VB10.16)
MSI mismatch-repair-derived peptides	Lynch-associated colorectal, endometrial, and related tumors	Recurrent tumor-created peptide classes in premalignant and malignant MSI tissue	Adenomas, advanced neoplasia, immune response, and eventually cancer incidence	Conceptual interception priority based on target biology; cancer-incidence reduction unproven; clinical prevention not yet shown. Lead platform example: heterologous viral-vector prime-boost (Nous-209: GAd20 + MVA) [36]
KRAS mutant peptides	PDAC, colorectal, lung, and selected other cancers	Recurrent driver mutations with HLA restriction	MRD-positive or high-risk adjuvant PDAC/CRC; ctDNA dynamics	Largest nonviral shared-driver family by population coverage; clinical efficacy of a shared mRNA construct is unproven. Platform examples: peptide-amphiphile (ELI-002; active), shared mRNA (V941; discontinued, illustrative platform reference only)
EBV latent antigens	Nasopharyngeal carcinoma; EBV-positive lymphomas and gastric carcinoma	Foreign viral antigens, but latency-pattern variability across diseases	Biomarker-confirmed EBV-positive measurable disease or MRD with checkpoint combinations	Latency-informed target selection essential; per-indication confirmation of antigen expression pattern and HLA presentation required. Platform example: shared mRNA (WGc-043; IND-cleared, no efficacy data)
IDH1 R132H	Diffuse IDH1-mutant glioma	Shared clonal mutation-derived class II epitope	Safety, immune response, progression measures, imaging	Strong target biology; specialized indication; clinical benefit not established. Lead platform: peptide vaccine (IDH1-vac; published phase 1)
H3 K27M	Diffuse midline glioma	Tumor-defining histone mutation	Small aggressive-disease trials with immune and radiographic readouts	High biological priority but rare and clinically complex; HLA-A*02:01 class I targeting is disputed. Lead platform: peptide vaccine (class II-focused; published early-phase data)
NPM1 mutant peptides	Acute myeloid leukemia	Insertion-altered C-terminal neoantigens; MRD measurable	Post-remission MRD and relapse prevention	Promising antigen with preclinical immunopeptidomic and TCR-engineering support; clinical feasibility and benefit untested; no pre-manufactured shared vaccine in late-stage clinical development
Mutant CALR exon 9	Myeloproliferative neoplasms	Frameshift-derived mutant C-terminus	Safety, mutant allele burden, symptom burden, progression markers	Promising antigen with preclinical peptide-vaccine support; clinical feasibility and benefit untested; early clinical exploration only
MCPyV T antigens	Virus-positive Merkel cell carcinoma	Foreign viral antigens; clinical maturity less developed than HPV	Aggressive measurable disease with PD-1 combination logic	Promising target biology; clinical maturity limited; no pre-manufactured shared product in late-stage clinical development
Personalized mRNA neoantigen (platform reference)	Resected high-risk melanoma; PDAC	Personalized, not shared, but proves mRNA platform can be clinically active in oncology	Recurrence-free survival in adjuvant setting	Platform reference only; not evidence for shared pre-manufactured vaccine success. Platform: personalized RNA (mRNA-4157/V940 KEYNOTE-942; autogene cevumeran)
BRCA-associated breast cancer	BRCA1/2 carriers and BRCA-related tumors	No validated universal shared tumor-only BRCA epitope	Discovery cohort sampling; tissue-restricted prevention trials	Risk-enrichment population for target discovery; not a current strict target. Probability that a broad BRCA library reaches practice is unknown and may be low. Platform status: research and discovery only

**Table 6:** Current-status table for 2024–2026 pipeline references. Source type indicates evidence tier; sponsor releases, company pipeline pages, trade press, and trial-registry entries are cited only for regulatory or trial-status facts and not as proof of clinical efficacy unless peer-reviewed outcome data exist. Status verified from the cited sources in June 2026.

Product	Target	Disease	Trial Identifier	Source Type	Latest Verified Status	What the Source Does Not Prove
BNT113	HPV16 E6/E7 (mRNA)	HPV16+ HNSCC	NCT04534205 (AHEAD-MERIT)	Sponsor release; trial registry	Fast Track designation; AHEAD-MERIT ongoing	No established clinical benefit; not approval
PDS0101	HPV16 (peptide)	HPV16+ R/M HNSCC	NCT06790966	Trial registry	Phase 3 ongoing with pembrolizumab	Non-mRNA comparator; no phase 3 outcome yet
VB10.16	HPV16 (DNA)	HPV16+ advanced cervical	NCT04405349	Peer-reviewed	Clinical signal with atezolizumab	Non-mRNA comparator; not definitive efficacy
BNT116	TAA (mRNA)	NSCLC	NCT05142189; NCT05557591	Sponsor release; trial registry	Phase 1 and Phase 2 development	No definitive efficacy reported
BNT111	Melanoma-associated antigens (mRNA, FixVac)	Advanced melanoma	NCT04526899	Sponsor release; trade press	Phase 2 topline; later trade-status uncertainty	Not a definitive success model
mRNA-4359	IDO1/PD-L1 (mRNA)	Advanced melanoma	NCT05533697	Sponsor release; trial registry; NCI dictionary	Phase 2 dose-expansion cohort within Phase 1/2; Fast Track (CPI-refractory)	Fast Track and first-line cohort are different populations; not proven benefit
EVM14	TAA (mRNA)	Squamous cell carcinomas	Not reported in cited source (global Phase I)	Sponsor release	Phase 1 first patient dosed (safety/tolerability)	No efficacy evidence
CVHNLC	Precision TAA (mRNA)	Squamous NSCLC	NCT07073183	Sponsor release	IND-cleared Phase 1 (safety/tolerability)	Investigational only; no efficacy
CVGBM	TAA epitopes (mRNA)	Glioblastoma	NCT05938387	Sponsor release	Phase 1 part B fully enrolled	Enrollment is not efficacy
WGc-043	EBV antigens (mRNA)	EBV-related cancers	Not reported in cited sources	Sponsor release; trade press	IND-cleared/allowed (US and China)	Not product-approved; no peer-reviewed efficacy
V941/mRNA-5671	KRAS G12D/G12V/G13D/G12C (mRNA)	NSCLC/CRC/PDAC	NCT03948763	Trial registry; trade press; NCI dictionary (mechanism)	Phase 1; program discontinued by partner	Historical; not an active pipeline asset
mRNA-4157/V940	Personalized neoantigens (mRNA)	Resected melanoma	NCT03897881 (KEYNOTE-942)	Peer-reviewed	RFS benefit with pembrolizumab	Personalized, not shared; no evidence for fixed-antigen products

**Table 7:** Validation gates and decision rules.

Gate	Evidence Required	Decision Rule
Tumor specificity	Tumor and normal-tissue immunopeptidome where available; transcript and protein expression; germline versus somatic status; T-cell cross-reactivity testing	Proceed only if a strict claim is defensible; otherwise reclassify as tumor-associated
Population recurrence and coverage	Prevalence by mutation, viral type, cancer site, HLA allele, stage; HLA frequency distribution across ancestral populations	Define the coverage claim precisely; report population breadth honestly
Natural presentation	HLA ligand detection on tumor cells; tumor-cell recognition by induced T cells; processing validation	Do not advance predicted-only epitopes as validated targets
Clonality and functional importance	Variant allele fraction; single-cell or bulk subclonal architecture; early-lesion data; dependence on antigen or driver pathway	Avoid subclonal passenger antigens when a shared product is intended
Safety	Normal-tissue recognition risk; autoimmunity potential; cytokine profile; neurologic or hematologic toxicity where relevant	Stop or reclassify if off-tumor risk is unacceptable; standards are stricter for prevention than for metastatic therapy
Clinical interpretability	MRD clearance, recurrence-free survival, objective response, adenoma reduction, ctDNA dynamics, or cancer incidence, chosen by setting	Do not interpret immune response as proof of prevention or survival benefit

**Table 8:** Target-by-target normal-tissue safety evidence and strict-rule classification. Entries summarize the current evidence the manuscript relies on; absence of reported normal-tissue presentation at current detection limits does not prove absolute absence, and each target requires confirmatory tumor and normal-tissue immunopeptidomics before clinical entry.

Target	Normal Transcript Expression	Normal Protein Expression	Normal Immunopeptidome Evidence	Cross-Reactivity Risk	Human Safety Data	Strict-Rule Classification
HPV16/18 E6/E7	Viral; absent from normal human genome	Absent from uninfected normal tissue; can be expressed in HPV-infected non-malignant epithelium, where eligibility is therefore defined by confirmed tumor or lesional viral-oncoprotein expression	Not expected in uninfected normal tissue (viral antigen)	Low; sequence is non-human	Large-scale prophylactic and therapeutic exposure; therapeutic E6/E7 in early trials	Conditional viral-oncoprotein target; eligibility requires confirmed tumor or lesional E6/E7 expression and absence of unacceptable normal-tissue risk
KRAS G12/G13	Wild-type KRAS ubiquitous; mutant restricted to tumor	Mutant neopeptide tumor-restricted	Mutant peptide–HLA reported on tumor; normal-tissue data incomplete	Wild-type cross-reactivity must be excluded per allele	Single-patient G12D adoptive-TCR regression; vaccine trials early	Strict tumor-specific at mutant residue; HLA-restricted
IDH1 R132H	Wild-type IDH1 broadly expressed; mutant tumor-restricted	Mutant neoepitope tumor-restricted	Class II presentation supported in glioma	Low if mutant-specific tile used	NOA-16 phase 1 safety and immunogenicity	Strict tumor-specific (mutation-derived)
H3 K27M	Wild-type H3.3 ubiquitous; mutant tumor-restricted	Mutant neoepitope tumor-restricted	Class II presentation supported; class I HLA-A*02:01 disputed	Low if mutant-specific tile used	Small early-phase/compassionate-use data	Strict tumor-specific (mutation-derived)
NPM1 Type A	Wild-type NPM1 broadly expressed; mutant tumor-restricted	Mutant C-terminus tumor-restricted	Mutant peptide directly identified on AML cells	Low; novel C-terminus	Preclinical/immunopeptidomic; clinical untested	Strict tumor-specific (insertion-altered C-terminus)
CALR exon 9	Wild-type CALR broadly expressed; mutant tumor-restricted	Mutant C-terminus tumor-restricted	MHC-I skewing reported; presentation context-dependent	Low; novel frameshift C-terminus	Preclinical peptide-vaccine; clinical early	Strict tumor-specific (frameshift C-terminus)
EWS-FLI1	Fusion junction absent from normal proteome	Junction peptide tumor-restricted	Not demonstrated for selected tile	Low for junction; presentation unproven	No vaccine-relevant T-cell recognition shown	Sequence-defined; not presentation-validated
BCR-ABL b3a2	Fusion junction absent from normal proteome	Junction peptide tumor-restricted	b3a2 peptide–HLA presentation supported	Low; artificial start M not part of natural junction	TKI therapy dominant; vaccine historic	Strict tumor-specific (fusion junction)
MCPyV LT/ST	Viral; absent from normal human genome	Absent from normal tissue	Not authenticated (placeholder)	Low; non-human	Less mature than HPV	Conceptual placeholder; not authenticated

**Table 9:** Construct-by-construct HLA restriction, expected population coverage, equity considerations, and validation status. Most allele-level immunopeptidomic and T-cell data derive from HLA contexts over-represented in cohorts of European descent (notably HLA-A*02:01); equitable library coverage requires adding and validating alleles relevant to the populations served. Coverage statements are qualitative and provisional. (**a**) Evidence tier, evidence basis, preferred early endpoint, and interpretive caveat by target family. Evidence tiers follow the two-tier scheme defined in the Conclusion (proof-of-mechanism versus proof-of-benefit). The tier and endpoint assignments reflect the authors’ synthesis of the evidence cited in the corresponding sections and are not formal rankings or predictions of regulatory success. Endpoints are stated per setting because immune response, ctDNA change, MRD clearance, and recurrence-free survival are not interchangeable. (**b**) Construct-by-construct restricting alleles with approximate allele or phenotype frequencies across major ancestry groups and the resulting coverage implication. Frequencies are drawn from large allele-frequency reference resources and published HLA surveys: values were verified against individual Allele Frequency Net Database (AFND) population entries [78] and cross-checked against the 200-population worldwide HLA survey of Arrieta-Bolaños et al. [79], the cross-sectional HLA-eligibility analysis of Olivier et al. [80] for landmark European and African HLA-A*02:01 values, and the high-resolution Eastern and Southern African class I survey of Banjoko et al. [81]; East Asian values reflect large Han Chinese and Vietnamese registry cohorts. Values are given as representative point estimates or narrow ranges from these named reference populations rather than exact figures for any single cohort, since allele frequencies vary by sub-population and reference dataset. Class II restriction for several mutation-derived tiles broadens coverage beyond the single class I allele shown. Empty or unresolved entries mark targets whose restriction is not yet mapped and therefore cannot be quantified. Where phenotype frequencies are unavailable, allele frequencies are used as approximations and should not be interpreted as direct patient-eligibility percentages.

Construct	Known/Expected HLA Restriction	Expected Population Coverage	Equity Consideration	Validation Status
HPV16/18 E6/E7	Multiple class I and class II alleles (broad epitope content)	Broad across populations	Relatively allele-agnostic; favorable for equity	Tumor expression verified; presentation per epitope to confirm
KRAS G12D	HLA-C*08:02 (documented), HLA-A*11:01 and others	Allele-dependent; gaps outside studied alleles	Coverage skews to studied alleles; needs broader allele validation	Single-patient proof of mechanism; per-allele validation needed
KRAS G12V/G12R/G12C/G13D	Allele-specific; partly characterized	Allele-dependent; incomplete mapping	Library must add alleles common in non-European ancestries	Predicted/partial; immunopeptidomic confirmation needed
IDH1 R132H	Class II (e.g., HLA-DR)	Moderate-broad via class II	Class II breadth aids coverage	Phase 1 immunogenicity; broader allele data limited
H3 K27M	Class II-restricted; class I HLA-A*02:01 disputed	Moderate via class II	HLA-A*02:01 over-studied; class II broadens reach	Early-phase; class I targeting unconfirmed
NPM1 Type A	Several class I/II alleles reported	Allele-dependent	Needs validation beyond index alleles	Preclinical/immunopeptidomic
CALR exon 9	Predominantly class II/CD4+ evidence; natural class I presentation is not established; coverage should not be expressed through a single class I allele frequency	Allele-dependent	Mapping incomplete across ancestries	Preclinical
EWS-FLI1	Not established for selected tile	Unknown	Cannot be assessed until presentation shown	Not presentation-validated
BCR-ABL b3a2	Defined class I presentation contexts	Allele-dependent	Mapping incomplete	Presentation supported; vaccine context historic
(**a**)				
**Target Family**	**Current Evidence tier**	**Evidence Basis for the Tier**	**Preferred Early Endpoint (and Setting)**	**Principal Interpretive Caveat**
HPV16/18 E6/E7	Proof-of-mechanism	Early-phase therapeutic E6/E7 immune-response and clinical-activity signals; large prophylactic and therapeutic exposure	Objective response or recurrence-free survival in antigen-positive measurable or adjuvant HNSCC and cervical/anal disease, often with checkpoint blockade	Strongest target biology, but no randomized therapeutic-vaccine benefit yet; prophylactic success does not equal therapeutic efficacy
MSI/Lynch frameshift peptides	Proof-of-mechanism	Single-arm phase 1b/2 immunogenicity in Lynch carriers (viral-vector platform); broad immunogenic-peptide validation	Adenoma or advanced-neoplasia incidence and immune response in Lynch carriers; cancer incidence over longer horizons	Interception model only; cancer-incidence reduction unproven; published platform is viral-vector, not mRNA
KRAS mutant peptides	Proof-of-mechanism	Single-patient G12D adoptive-TCR regression; shared-peptide (non-mRNA) MRD trial with biomarker-defined RFS signal	ctDNA dynamics and RFS in MRD-positive or high-risk adjuvant PDAC and CRC	Shared mRNA construct unproven; HLA-restricted; strongest shared-peptide data are peptide-amphiphile, not mRNA
IDH1 R132H	Proof-of-mechanism	Phase 1 safety and high-frequency class II immune responses (non-randomized; peptide platform)	Immune response, progression measures, and imaging in IDH1-mutant glioma	Mechanistic basis only; no randomized survival benefit; specialized indication
H3 K27M	Proof-of-mechanism	Early-phase safety and mutation-specific T-cell responses, including a durable case (peptide platform)	Immune and radiographic readouts in small diffuse-midline-glioma cohorts	Rare, aggressive disease; class I HLA-A*02:01 targeting disputed; class II focus
NPM1 mutant peptides	Proof-of-mechanism (preclinical-weighted)	Direct immunopeptidomic detection on AML cells; preclinical TCR-engineering cytotoxicity	Post-remission MRD kinetics and relapse prevention in NPM1-mutant AML	No pre-manufactured shared vaccine in late-stage development; clinical benefit untested
CALR exon 9	Proof-of-mechanism (preclinical-weighted)	Shared-neoantigen identification; preclinical optimized peptide-vaccine data; early clinical exploration	Mutant allele burden, symptom burden, and progression markers in CALR-mutant MPN	Early exploration only; presentation context-dependent; clinical benefit untested
MCPyV T antigens	Conceptual proof-of-mechanism rationale	Viral-antigen rationale analogous to HPV; clinical maturity limited; placeholder construct only	Measurable-disease response with PD-1 combination in virus-positive MCC	No sequence-anchored construct authenticated here; no late-stage shared product
EBV latent antigens	Conceptual proof-of-mechanism rationale	Foreign viral antigens; one IND-cleared mRNA candidate without efficacy data	Biomarker-confirmed EBV-positive measurable disease or MRD with checkpoint combinations	Latency-pattern variability requires per-indication confirmation; no efficacy data
EWS-FLI1/fusion junctions	Sequence-defined (below proof-of-mechanism)	Fusion structure and transcript authenticated; natural presentation of the selected tile not demonstrated	Presentation and tumor-recognition assays before any clinical endpoint	Not presentation-validated; breakpoint heterogeneity limits a single construct
BCR-ABL b3a2	Proof-of-mechanism (historic)	Direct immunopeptidomic evidence of b3a2 presentation on leukemic cells	Largely superseded; immune response if revisited in TKI-resistant settings	TKI therapy dominant; vaccine development historic
Personalized mRNA neoantigen (platform reference)	Proof-of-benefit (platform only)	Randomized phase 2b RFS benefit with pembrolizumab in resected melanoma	Recurrence-free survival in the adjuvant setting	Personalized, not shared; does not establish benefit for fixed-antigen products
BRCA-associated breast cancer	Below proof-of-mechanism (discovery only)	No validated shared tumor-only epitope; exploratory computational candidates only	Somatic-target discovery and normal-tissue safety in risk-enriched cohorts	Risk marker, not antigen; probability of a broad library reaching practice is unknown and may be low
(**b**)				
**Construct**	**Restricting** **Allele(s) Cited or Expected**	**Approx.** **Frequency,** **European Ancestry**	**Approx.** **Frequency, East Asian Ancestry**	**Approx.** **Frequency,** **African** **Ancestry**
KRAS G12D (documented)	HLA-C*08:02	~5 to 6%	~1 to 2%	~6 to 8%
KRAS G12D/hotspots (additional)	HLA-A*11:01	~6%	~22 to 27%	~3 to 6%
KRAS hotspots (broad class I)	HLA-A*02:01 and others	~27%	~9 to 12%	~12%
IDH1 R132H	Class II (HLA-DR, including DRB1*01:01-associated presentation)	DRB1*01:01 ~10 to 11%; class II presentation broader	Lower DRB1*01:01; other DR alleles relevant	Variable; other DR alleles relevant
H3 K27M	Class II-restricted; class I HLA-A*02:01 disputed	A*02:01 ~27% (disputed for this tile)	A*02:01 ~9 to 12%	A*02:01 ~12%
NPM1 Type A	Several class I/II alleles reported (e.g., HLA-A*02:01 context)	~27% via A*02:01 context	~9 to 12%	~12%
CALR exon 9	Predominantly class II-restricted (CD4+); no established class I presenting allele [82,83]	Class II-restricted; class I frequency not the relevant metric	Class II-restricted; class I frequency not the relevant metric	Class II-restricted; class I frequency not the relevant metric
BCR-ABL b3a2	HLA-A*03:01 (direct immunopeptidomic evidence [84]); high-affinity binding also to A*11 and B*08 [85]; class II contexts (DRB1*01:01, *04:01, *09:01) reported	A*03:01 ~14% (0.140)	A*03:01 ~1.4% (0.014)	A*03:01 ~8% (0.084)
HPV16/18 E6/E7	Multiple class I and class II alleles (broad epitope content)	Broad	Broad	Broad
EWS-FLI1	Not established for the selected tile	Unknown	Unknown	Unknown

**Table 10:** Clinical development models by target class.

Target Class	Best First Clinical Setting	Most Relevant Endpoints	Representative Examples
Viral oncoproteins	Antigen-positive measurable or adjuvant disease, often with checkpoint blockade	Response, PFS, recurrence-free survival, antigen-specific T cells	HPV16-positive HNSCC (shared mRNA: BNT113; peptide: ISA101; DNA: VB10.16); cervical and anal cancer; virus-positive Merkel cell carcinoma (investigational); EBV-positive nasopharyngeal carcinoma (shared mRNA: WGc-043, IND-cleared)
Driver neoepitopes	Mutation-positive and HLA-compatible MRD or high-risk adjuvant disease	ctDNA, RFS, immune response, tumor recognition	KRAS-mutant PDAC and CRC (peptide-amphiphile: ELI-002 AMPLIFY-201; personalized RNA: autogene cevumeran); IDH1 glioma (peptide: IDH1-vac); H3 K27M glioma (peptide)
Mismatch-repair-derived peptide panels	High-risk inherited or molecularly defined MSI setting	Safety, immune response, adenoma biology, cancer incidence over time	Lynch syndrome (viral-vector prime-boost: Nous-209 GAd20 + MVA) [36]; selected MSI cancers
Hematologic neoantigens	Remission or molecular residual disease	MRD kinetics, relapse-free survival, serial immune monitoring	NPM1-mutated AML (preclinical immunopeptidomic; TCR engineering); CALR-mutant MPN (preclinical peptide-vaccine evidence)
Local immune therapy	Anatomically accessible tumor field with repeat access	Recurrence, progression, complete response, cystectomy-free survival	Non-muscle-invasive bladder cancer (live attenuated bacterium: BCG; cytokine + BCG: Anktiva plus BCG)
Tissue-restricted antigens	High-risk cohort with explicit safety tradeoff	Safety, tissue effects, immune response, recurrence biomarkers	Alpha-lactalbumin prevention concepts (peptide); MUC1 adenoma studies (peptide)
BRCA risk without validated epitope	Discovery and translational cohorts; not universal vaccination of unaffected carriers	Somatic target discovery, normal-tissue safety, early-lesion biology	BRCA1 and BRCA2 carriers (research and discovery only)

**Table 11:** Target-passport template for a library module. A module without a complete passport is a discovery inventory entry, not a clinically applicable library member. Fields requiring evidence should record the evidence tier and citation, or explicitly state that the evidence is not yet available.

Passport Field	Required Entry
Module identifier	Unique library ID and version number
Antigen class	Viral oncoprotein, driver neoepitope, frameshift peptide, insertion-altered C-terminus, or fusion junction
Source accession and version	UniProtKB or NCBI RefSeq accession with version and retrieval date
Isoform/source residue range	Isoform used and the exact source amino-acid range
Precise molecular event	Mutation, deletion, substitution, frameshift, or junction applied, with coordinates
Expected translated product	Final encoded protein sequence and ORF, with construct length
ORF checksum	MD5 (or equivalent) checksum of the final ORF for reproducibility
Tumor type/risk syndrome	Defined molecular subgroup or inherited-risk context
HLA restriction	Known or expected restricting alleles, or stated as unestablished
Normal-tissue evidence	Transcript, protein, and immunopeptidome evidence of absence from essential normal tissue
Natural-presentation evidence	Immunopeptidomic detection on tumor cells, or stated as not demonstrated
T-cell recognition evidence	Direct tumor-cell recognition by induced T cells, or stated as not demonstrated
Clinical evidence tier	Proof-of-mechanism or proof-of-benefit, with citation
Safety evidence	Normal-tissue cross-reactivity, autoimmunity, and organ-toxicity data
Escape risk and monitoring	Clonality, antigen-loss risk, and escape-monitoring plan
Preferred clinical setting	Adjuvant, MRD, measurable disease, interception, or prevention
Combination strategy	Checkpoint blockade or other immune modulation as applicable
Retirement trigger	Pre-specified conditions that downgrade or remove the module

**Table 12:** Module-retirement decision tree. The steps are applied sequentially to each library module as new evidence accrues; a module advances only while each gate is satisfied, and any pre-specified retirement trigger removes or downgrades it. A living target atlas governed by these rules is more realistic than a fixed universal construct.

Step	Decision Question	If Yes	If No
1	Is the target still absent from essential normal tissues at the peptide–HLA level as detection methods improve?	Proceed to Step 2	Downgrade or retire; reclassify as tumor-associated
2	Is the target naturally presented on tumor cells (immunopeptidomics or tumor-cell T-cell recognition)?	Proceed to Step 3	Downgrade to sequence-defined; suspend clinical use pending presentation data
3	Does population coverage remain adequate after HLA-frequency filtering for the intended population?	Proceed to Step 4	Restrict indication or add alleles; flag equity gap
4	Is the target clonal and resistant to antigen loss under immune pressure?	Proceed to Step 5	Downgrade if frequent escape; consider multi-epitope or combination design
5	Is there a clinical signal (or acceptable rationale) despite adequate immunogenicity?	Retain as active module; schedule re-review	Downgrade; retain only if disease is aggressive and target is uniquely clean
6	Have any pre-specified retirement triggers been met (cross-reactivity, toxicity, loss of coverage, persistent absence of benefit)?	Retire or remove module; document rationale and version	Retain; continue routine surveillance and periodic re-review

**Table 13:** Source-anchoring summary for the sequence-anchored constructs and the MCPyV placeholder. Each construct is anchored by a canonical component accession together with the applied mutation, frameshift, or fusion-junction definition; protein-accession identity alone does not authenticate the mutant, frameshift, or fusion constructs. Verification status refers to the two-stage computational check described above.

Construct	Canonical Component Accession(s)	Transcript/Exon Coordinates	Mutation or Junction Event	Resulting Key Peptide Feature	Literature Source	Verification Status
HPV16 E6	UniProt P03126	full E6 ORF; C-terminus	Deletion of residues 152–158 (TRRETQL); removes ETQL PDZ motif	Attenuated (PDZ-deleted) E6 antigenic reference design retaining T-cell epitopes	[87,88]	Verified (Stage 1 + 2)
HPV16 E7	UniProt P03129	full E7 ORF; LXCXE motif	C24G substitution (LXCXE)	Attenuated (LXCXE-disrupted) E7 antigenic reference design retaining T-cell epitopes	[89]	Verified (Stage 1 + 2)
HPV18 E6	UniProt P06463	full E6 ORF; C-terminus	Deletion of residues 153–158 (RRETQV); removes ETQV PDZ motif	Attenuated (PDZ-deleted) E6 reference design	[87,88]	Verified (Stage 1 + 2)
HPV18 E7	UniProt P06788	full E7 ORF; LXCXE motif	LXCXE cysteine to glycine substitution	Attenuated (LXCXE-disrupted) E7 reference design	[89]	Verified (Stage 1 + 2)
KRAS G12D/G12V/G12R/G12C	UniProt P01116	KRAS codons 12–13 (exon 2)	Single-codon missense at G12 or G13	Mutation-spanning minigene	[17,49,50]	Verified (Stage 1 + 2)
KRAS G13D	UniProt P01116	KRAS codon 13 (exon 2)	G13D missense	Mutation-spanning minigene	[49,50]	Verified (Stage 1 + 2)
IDH1 R132H	UniProt O75874	IDH1 codon 132; tile p123–142	R132H missense	Class II-restricted 20-mer (IDH1-vac)	[59,60]	Verified (Stage 1 + 2)
H3 K27M	UniProt P84243	H3.3 codon 28 (clinical K27); tile aa 22–41	K27M (canonical residue 28) missense	Class II-restricted tile	[61,90,91]	Verified (Stage 1 + 2)
NPM1 Type A	UniProt P06748	exon 12; aa 251–298	+4 bp insertion; WQWRKSL to CLAVEEVSLRK	Insertion-altered novel C-terminus	[19,92]	Verified (Stage 1 + 2)
CALR exon 9 (shared)	UniProt P27797	exon 9; +1 frameshift	Shared 36-aa novel C-terminus (type 1 del52/type 2 ins5 converge)	CALRLong36 neoantigen	[18,93]	Verified (Stage 1 + 2)
EWS-FLI1 Type 1	RefSeq NP_001157610.1; NP_002008.1	EWSR1 exon 7 to FLI1 exon 6; t(11;22)(q24;q12)	Type 1 fusion junction	Junction-spanning 21-mer	[94,95]	Verified (Stage 1 + 2)
BCR-ABL b3a2	RefSeq NP_004318.3; NP_005148.2	BCR e14 to ABL1 exon 2; t(9;22)(q34;q11)	b3a2 (p210) fusion junction	Junction-spanning 17-mer	[84]	Verified (Stage 1 + 2)
MCPyV LT/ST	UniProt B6DVY9; B0G0V7	not finalized	Conceptual attenuating substitutions	No sequence-anchored ORF provided	[16,96]	Conceptual placeholder; not authenticated

**Table 14:** Sequence-level verification status for the fifteen sequence-anchored candidates and the one conceptual placeholder. All sequence-anchored candidates pass both stages of computational verification: (Stage 1) the claimed encoded protein matches the canonical UniProt/RefSeq record with the documented mutation, attenuation, or literature-defined frameshift or junction sequence applied; (Stage 2) the ORF translates to the claimed protein exactly under the standard human genetic code. (**a**) Separation of sequence correctness from biological validation for each construct. The fourth column reports residue-level confirmation that the translated ORF matches the claimed protein (the scope of the computational check); the fifth column reports, separately, whether natural peptide–HLA presentation has been experimentally demonstrated. Sequence correctness does not imply demonstrated presentation, and entries marked as not demonstrated or predicted remain to be validated by tumor and normal-tissue immunopeptidomics or direct T-cell recognition before clinical entry.

Candidate	Source Accession	Encoded Antigenic Region	Construct Length	Status
1a. HPV16 E6	UniProt P03126	E6 residues 1–151 (C-terminal PDZ motif TRRETQL, residues 152–158, deleted)	456 bp/151 aa	Sequence-anchored; verified
1b. HPV16 E7	UniProt P03129	E7 residues 1–98 with C24G substitution only (LXCXE attenuation)	297 bp/98 aa	Sequence-anchored; verified
1c. HPV18 E6	UniProt P06463	E6 residues 1–152 (C-terminal PDZ motif RRETQV, residues 153–158, deleted)	459 bp/152 aa	Sequence-anchored; verified
1d. HPV18 E7	UniProt P06788	E7 residues 1–105 with C27G substitution only (LXCXE attenuation)	318 bp/105 aa	Sequence-anchored; verified
2. MCPyV LT/ST	UniProt B6DVY9 (LT); B0G0V7 (ST)	LT and ST antigenic regions with attenuating substitutions	Not finalized	Conceptual placeholder; design specification only
3. KRAS G12D	UniProt P01116	KRAS residues 1–37 with G12D	114 bp/37 aa	Sequence-anchored; verified
4. KRAS G12V	UniProt P01116	KRAS residues 1–37 with G12V	114 bp/37 aa	Sequence-anchored; verified
5. KRAS G12R	UniProt P01116	KRAS residues 1–37 with G12R	114 bp/37 aa	Sequence-anchored; verified
6. KRAS G12C	UniProt P01116	KRAS residues 1–37 with G12C	114 bp/37 aa	Sequence-anchored; verified
7. KRAS G13D	UniProt P01116	KRAS residues 1–37 with G13D	114 bp/37 aa	Sequence-anchored; verified
8. IDH1 R132H	UniProt O75874	Artificial M + IDH1 residues 123–142 with R132H	66 bp/21 aa	Sequence-anchored; verified
9. H3 K27M	UniProt P84243	Artificial M (replacing L21) + H3.3 residues 22–41 with K28M	66 bp/21 aa	Sequence-anchored; verified
10. NPM1 Type A	UniProt P06748	Artificial use of the natural internal Met at residue 251; wild-type residues 251–287 followed by the 11-aa novel mutant C-terminus CLAVEEVSLRK (residues 288–298 of the mutant protein) generated by the Type A +4 insertion in exon 12, replacing the wild-type C-terminal WQWRKSL (residues 38–48 of the encoded tile)	147 bp/48 aa	Sequence-anchored; verified
11. CALR (shared C-terminus)	UniProt P27797	Artificial M + shared 36-aa novel mutant C-terminus common to type 1 (c.1099_1150del52) and type 2 (c.1154_1155insTTGTC) CALR mutations	114 bp/37 aa	Sequence-anchored; verified
12. EWS-FLI1 Type 1	RefSeq NP_001157610.1 + NP_002008.1	Artificial M + 21-aa EWS-FLI1 Type 1 junction (EWSR1 exon 7 + FLI1 exon 6)	69 bp/22 aa	Sequence-anchored; verified
13. BCR-ABL b3a2	RefSeq NP_004318.3 (BCR) + NP_005148.2 (ABL1)	Artificial M + 17-aa BCR-ABL b3a2 junction (BCR exon e14 + ABL1 exon 2)	57 bp/18 aa	Sequence-anchored; verified
(**a**)				
**Construct**	**Canonical** **Accession**	**Mutant/Junction/** **Attenuation Event**	**Translated ORF** **Residue-Level** **Confirmed?**	**Natural Peptide–HLA Presentation Demonstrated?**
HPV16 E6	UniProt P03126	PDZ-motif deletion (residues 152 to 158)	Yes (Stage 1 and 2)	Viral oncoprotein presented in HPV-positive tumors; per-epitope presentation of the attenuated construct to be confirmed
HPV16 E7	UniProt P03129	C24G (LXCXE) substitution	Yes (Stage 1 and 2)	E7-derived peptide–HLA presentation supported in HPV-positive disease; construct-specific confirmation pending
HPV18 E6	UniProt P06463	PDZ-motif deletion (residues 153 to 158)	Yes (Stage 1 and 2)	As for HPV16 E6; construct-specific confirmation pending
HPV18 E7	UniProt P06788	C27G (LXCXE) substitution	Yes (Stage 1 and 2)	As for HPV16 E7; construct-specific confirmation pending
KRAS G12D	UniProt P01116	G12D missense	Yes (Stage 1 and 2)	Yes for HLA-C*08:02 (single-patient adoptive-TCR regression); other alleles require confirmation
KRAS G12V/G12R/G12C	UniProt P01116	G12V/G12R/G12C missense	Yes (Stage 1 and 2)	Partial/predicted; allele-resolved immunopeptidomic confirmation needed
KRAS G13D	UniProt P01116	G13D missense	Yes (Stage 1 and 2)	Predicted/partial; confirmation needed
IDH1 R132H	UniProt O75874	R132H missense	Yes (Stage 1 and 2)	Class II presentation supported in glioma (clinical immunogenicity)
H3 K27M	UniProt P84243	K27M (canonical residue 28) missense	Yes (Stage 1 and 2)	Class II presentation supported; class I HLA-A*02:01 presentation reported but not confirmed in vivo
NPM1 Type A	UniProt P06748	+4 bp insertion; WQWRKSL to CLAVEEVSLRK	Yes (Stage 1 and 2)	Yes, mutant peptide directly identified on AML cells by immunopeptidomics
CALR exon 9 (shared)	UniProt P27797	Shared 36-aa novel C-terminus (type 1/type 2 converge)	Yes (Stage 1 and 2)	Shared neoantigen recognized by T cells; presentation context-dependent (MHC-I skewing reported)
EWS-FLI1 Type 1	RefSeq NP_001157610.1 + NP_002008.1	EWSR1 exon 7 to FLI1 exon 6 junction	Yes (Stage 1 and 2)	No, natural presentation of the selected junction tile not demonstrated (sequence-defined only)
BCR-ABL b3a2	RefSeq NP_004318.3 + NP_005148.2	b3a2 (p210) junction	Yes (Stage 1 and 2)	Yes, b3a2 junction peptide–HLA presentation directly evidenced on leukemic cells
MCPyV LT/ST	UniProt B6DVY9 + B0G0V7	Conceptual attenuating substitutions	No (no finalized ORF; placeholder)	Not authenticated; conceptual placeholder only

**Table 15:** Verification URLs for source accessions.

Candidate	Source Accession	Verification URL
HPV16 E6	UniProt P03126	https://www.uniprot.org/uniprotkb/P03126 (access on 6 June 2026)
HPV16 E7	UniProt P03129	https://www.uniprot.org/uniprotkb/P03129 (access on 6 June 2026)
HPV18 E6	UniProt P06463	https://www.uniprot.org/uniprotkb/P06463 (access on 6 June 2026)
HPV18 E7	UniProt P06788	https://www.uniprot.org/uniprotkb/P06788 (access on 6 June 2026)
MCPyV large T (design specification only)	UniProt B6DVY9	https://www.uniprot.org/uniprotkb/B6DVY9 (access on 6 June 2026)
MCPyV small T (design specification only)	UniProt B0G0V7	https://www.uniprot.org/uniprotkb/B0G0V7 (access on 6 June 2026)
KRAS (all hotspot variants)	UniProt P01116	https://www.uniprot.org/uniprotkb/P01116 (access on 6 June 2026)
IDH1	UniProt O75874	https://www.uniprot.org/uniprotkb/O75874 (access on 6 June 2026)
Histone H3.3, encoded by H3-3A/H3F3A and H3-3B/H3F3B	UniProt P84243	https://www.uniprot.org/uniprotkb/P84243 (access on 6 June 2026)
NPM1	UniProt P06748	https://www.uniprot.org/uniprotkb/P06748 (access on 6 June 2026)
CALR	UniProt P27797	https://www.uniprot.org/uniprotkb/P27797 (access on 6 June 2026)
EWSR1 (EWS-FLI1 fusion component)	NCBI RefSeq NP_001157610.1	https://www.ncbi.nlm.nih.gov/protein/NP_001157610.1 (access on 6 June 2026)
FLI1 (EWS-FLI1 fusion component)	NCBI RefSeq NP_002008.1	https://www.ncbi.nlm.nih.gov/protein/NP_002008.1 (access on 6 June 2026)
BCR (BCR-ABL fusion component)	NCBI RefSeq NP_004318.3	https://www.ncbi.nlm.nih.gov/protein/NP_004318.3 (access on 6 June 2026)
ABL1 (BCR-ABL fusion component)	NCBI RefSeq NP_005148.2	https://www.ncbi.nlm.nih.gov/protein/NP_005148.2 (access on 6 June 2026)

## Data Availability

The original contributions presented in this study are included in the article. Further inquiries can be directed to the corresponding author.
